# Co‐design development of a decision guide on eating and drinking for people with severe dementia during acute hospital admissions

**DOI:** 10.1111/hex.13672

**Published:** 2023-01-17

**Authors:** Kanthee Anantapong, Andrea Bruun, Anne Walford, Christina H. Smith, Jill Manthorpe, Elizabeth L. Sampson, Nathan Davies

**Affiliations:** ^1^ Marie Curie Palliative Care Research Department, Division of Psychiatry University College London London UK; ^2^ Department of Psychiatry, Faculty of Medicine Prince of Songkla University Hat Yai Thailand; ^3^ Faculty of Health, Science, Social Care and Education Kingston University London UK; ^4^ Family Carer Patient and Public Involvement Panel London UK; ^5^ Language and Cognition, Division of Psychology and Language Sciences University College London London UK; ^6^ NIHR Policy Research Unit in Health & Social Care Workforce, King's College London London UK; ^7^ NIHR Applied Research Collaboration (ARC) South London London UK; ^8^ Department of Psychological Medicine Royal London Hospital, East London NHS Foundation Trust London UK; ^9^ Research Department of Primary Care and Population Health, Centre for Ageing Population Studies University College London London UK

**Keywords:** carers, co‐design, decision aid, dementia, hospital care, hydration, nutrition

## Abstract

**Introduction:**

Using co‐design processes, we aimed to develop an evidence‐based decision guide for family carers and hospital professionals to support decision‐making about eating and drinking for hospital patients with severe dementia.

**Methods:**

Following a systematic review, we interviewed people with mild dementia, family carers and hospital professionals in England. We then held co‐design workshops with family carers and hospital professionals. In parallel with the workshops, we used a matrix to synthesize data from all studies and to develop a decision guide prototype. The prototype was iteratively refined through further co‐design workshops and discussions among researchers and Patient and Public Involvement (PPI) representatives. We conducted user testing for final feedback and to finalize the decision guide.

**Results:**

Most participants acknowledged the limited benefits of tube feeding and would not use or want it for someone with severe dementia. However, they found decision‐making processes and communication about nutrition and hydration were emotionally demanding and poorly supported in acute hospitals. The co‐design groups developed the aims of the decision guide to support conversations and shared decision‐making processes in acute hospitals, and help people reach evidence‐based decisions. It was designed to clarify decision‐making stages, provide information and elicit the values/preferences of everyone involved. It encouraged person‐centred care, best‐interests decision‐making and multidisciplinary team working. From user testing, family carers and hospital professionals thought the decision guide could help initiate conversations and inform decisions. The final decision guide was disseminated and is being used in clinical practice in England.

**Conclusion:**

We used rigorous and transparent processes to co‐design the decision guide with everyone involved. The decision guide may facilitate conversations about nutrition and hydration and help people reach shared decisions that meet the needs and preferences of people with severe dementia. Future evaluation is required to test its real‐world impacts.

**Patient or Public Contribution:**

People with mild dementia, family carers and hospital professionals contributed to the design of the decision guide through the interviews and co‐design workshops. PPI members helped design study procedures and materials and prepare this manuscript.

## 1 INTRODUCTION

In the severe stages, people living with dementia often develop eating and drinking problems, for example, swallowing difficulties, holding food in the mouth, changes in appetite and other changes in eating habits.^1^, ^2^ These eating and drinking problems may worsen during acute hospital admissions where people have to adapt to strict and busy hospital routines,^3^, ^4^ and their personal care, including help with eating and drinking, can be suboptimal.^5^ Although there is no evidence regarding the benefits of tube feeding for people with severe dementia^6^ and it is rare in the United Kingdom, they may still receive futile interventions, resulting from the influences of cultural beliefs, legal restrictions and family requests.^7^, ^8^, ^9^

With the declining capacity of the person with severe dementia to make decisions, decisions in hospitals about their nutrition and hydration are usually made by healthcare professionals with input from family carers, under the Mental Capacity Act (England and Wales).^10^, ^11^, ^12^ People with mild dementia may also have difficulty discussing their wishes and decisions^13^, ^14^ and want to leave future decisions about eating and drinking to family carers and professionals.^15^ However, it is still unclear how such decision‐making can be best supported, and both family carers and professionals often find making these decisions challenging.^8^, ^16^, ^17^

Decision aids or guides (henceforth decision guide(s)) are tools developed to help people participate in decision‐making about healthcare options, clarify and communicate their personal values and promote deliberation between everyone involved about options.^18^ Studies report people who had used a decision guide had more opportunities to discuss care decisions with their clinician, had more accurate risk perceptions and participated more in decision‐making.^19^

From our previous studies, decision guides regarding care for people with severe dementia, including at the end‐of‐life, seemed to be helpful^20^, ^21^, ^22^, ^23^, ^24^, ^25^; however, the earlier guides have been developed for either practitioners or family carers to make decisions for people with severe dementia and not specific to eating and drinking issues in acute hospitals, which are particularly challenging. This co‐design study, therefore, aimed to develop a decision guide covering nutrition and hydration for people with severe dementia in acute hospitals. The decision guide was expected to be used together by family carers and hospital professionals in making difficult decisions. The specific research objectives were to:

1. Using a matrix approach, synthesize data from a systematic review and co‐design workshops and interviews with people with mild dementia, family carers and hospital professionals;
2. Understand problems and possible solutions around eating and drinking decisions for people with severe dementia in acute hospitals;
3. Identify the focus and aims of the decision guide;
4. Map the problems and possible solutions identified from the synthesis into the decision guide;
5. Identify suitable format, content and dissemination of the decision guide.

## 2 METHODS

In developing the decision guide, we used a co‐design approach, in which researchers work in partnership with end users to gain deeper insights into the world of research participants and develop research outputs that are based on their experiences, perspectives and needs.^26^, ^27^ We based our co‐design processes on the 2005 Double Diamond design model proposed by the British Design Council which involves ‘discovering’ problems and needs of the users, ‘defining’ the challenges and areas to focus upon, ‘developing’ potential solutions and ‘delivering’ solutions that work.^28^ The co‐design research involves a range of methods, including interviews, surveys, focus groups or workshops,^29^, ^30^ and can be used to produce a variety of outputs, such as theoretical frameworks, commissioning statements, documents and interventions.^29^ It has been used to co‐design many healthcare interventions, including decision tools in dementia and palliative care.^24^, ^31^

**Figure 1:**
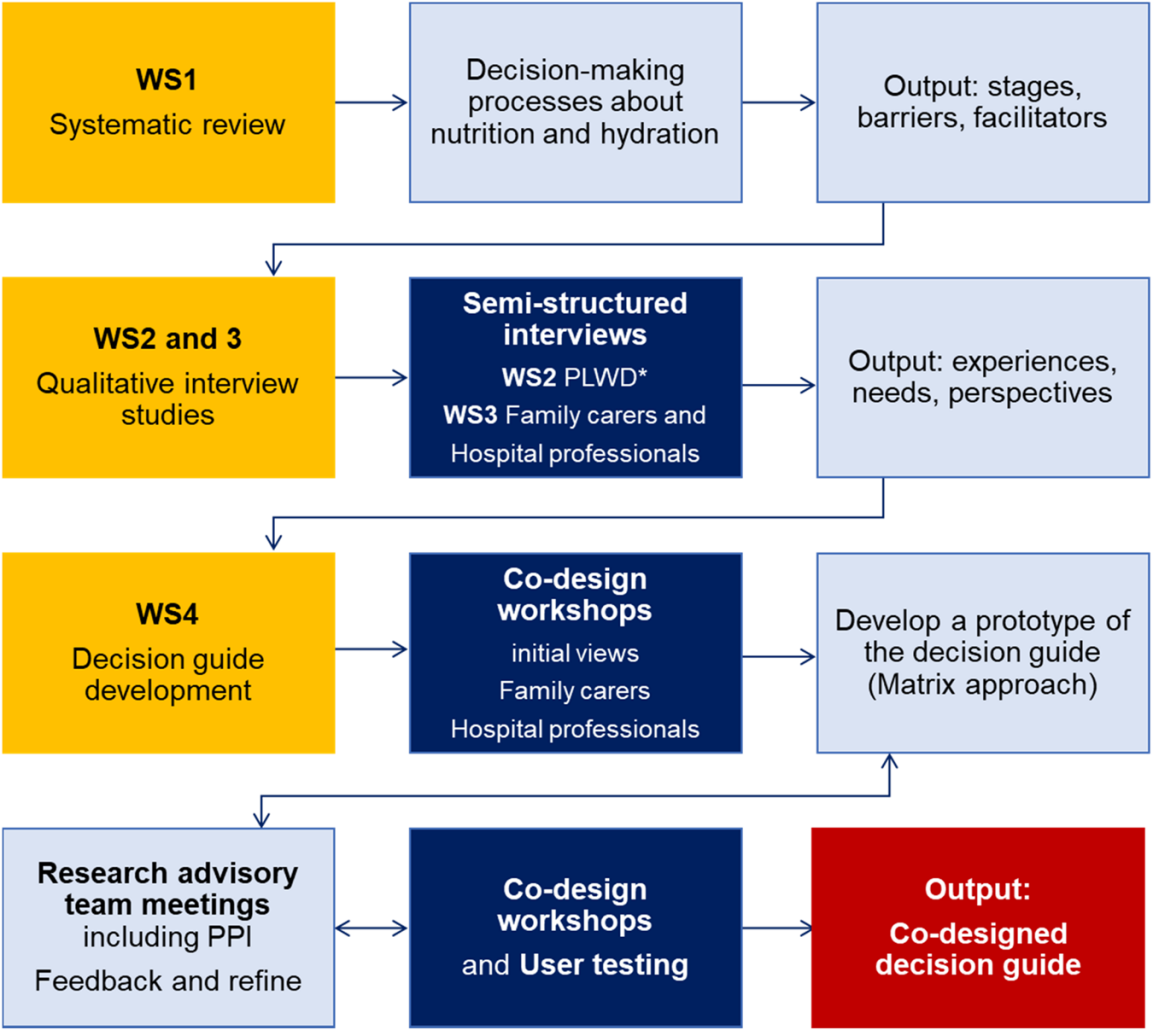
Diagram shows the sequence and outputs of all workstreams in the co‐design processes. *PLWD, people living with dementia.

As shown in Figure 1, the co‐design processes in this study comprised four sequential workstreams; Workstream 1 (WS1): a systematic review of decision‐making processes about nutrition and hydration for people with dementia^8^; WS2: semistructured interviews with people with mild dementia about their perspectives regarding future possible eating and drinking problems^15^; WS3: semistructured interviews with family carers and hospital professionals about care for nutrition and hydration for people with severe dementia during acute hospital admissions^32^ and WS4: a co‐design workshop to develop a decision guide about nutrition and hydration for a patient with severe dementia in an acute hospital. WS1–3 have been reported in separate publications, and this article focuses on the last WS4.

### 2.1 Population and participants

In WS4, we screened potential participants against the eligibility criteria in Table 1.

**Table 1:** Eligibility criteria for participants in this study

Family carers
Inclusion criteria
Family member or friend who is/was a key decision‐maker for a person with severe dementia (current or bereaved/former)
Participants must be able to provide informed consent
Participants must be able to read and speak English
Participants must be over the age of 18 years
Exclusion criteria
Family carers bereaved in the past 3 months
Hospital professionals
Inclusion criteria
Hospital professionals in a caring role, either health or social care, for people with severe dementia
Experienced in providing dementia care and contributing to decision‐making related to nutrition and hydration in acute hospital setting
Participants must be able to provide informed consent
Participants must be able to read and speak English

Due to limited time, financial support and accessibility caused by COVID‐19, we did not include people with dementia as participants in the online co‐design workshops (WS4). We appreciated that online workshops with people with dementia would require far more complex and sensitive processes.^33^ For example, ongoing mental capacity assessments and constant training and technical support, which need adjustments to the individual's changing capabilities and needs as dementia progresses. However, we included the experiences and needs of people with mild dementia identified from WS2 interviews throughout the co‐design processes and development of our decision guide.

### 2.2 Participant recruitment and consent process

Due to COVID‐19, we recruited family carers from Join Dementia Research (JDR) and online social media. JDR is an online self‐registration service that enables volunteers with memory problems or dementia, family carers of those with memory problems or dementia and healthy volunteers to register their interest in taking part in the research.

We recruited hospital professionals (henceforth professionals for brevity) using online social media. Potential professional participants were also invited via contacts of the research team and snowballing methods.^34^ As a result, few professional participants had a prior work‐related relationship with the research team, but we monitored its influence on research processes and reassured the participants about voluntariness and confidentiality. To obtain rich information, we purposively sampled professionals from the range of roles that would be part of a multidisciplinary team providing care for people with severe dementia.

We also invited family carers and professionals who had participated in the WS3 interviews who had expressed their interest in subsequent studies. Participant information sheets and consent forms were sent to potential participants who were given sufficient time to consider the study. We obtained written consent from participants before the workshops.

### 2.3 Research advisory team meetings

We held three research advisory team meetings to discuss the interpretation of discussions from the co‐design workshops and feedback on the prototype. They consisted of research team members and a Patient and Public Involvement (PPI) member, who was a family carer. The research team comprised old age and consultation liaison psychiatrists, a psychologist, a social care researcher, a speech and language therapist, a gerontologist and a linguist. We shared our general views that palliative care for older people with dementia should focus on the quality of life and avoid futile interventions. This may impact our interpretation of data generated in this study and the development of our decision guide. However, we reflected on our assumptions and involved a PPI member in providing feedback on the interpretations and development throughout the research processes.

### 2.4 Data collection methods

#### 2.4.1 Contributions from the earlier workstreams to the design of this study

The systematic review (WS1) informed the structure of case scenarios used in the discussions in co‐design workshops. Interview findings with people with mild dementia, family carers and professionals (WS2–3) were fed into the co‐design process, in terms of identifying problems and needs, providing feedback and solutions and generating ideas.^26^, ^30^, ^35^ We also asked interview participants for suggestions about the design of our decision guide.

In summary, from WS2, people with mild dementia want to postpone discussions about future eating and drinking problems, partly due to fears of being a burden to family and of being treated like a child. They wish to maintain a good quality of life rather than be kept alive at later stages by artificial nutrition and hydration.^15^ In WS3, family carers often experienced frustration with delays and repeated conversations about eating and drinking with different professionals, while professionals felt unprepared for decision‐making and found it challenging to work across the multidisciplinary team.^32^ In particular, as people with dementia were not included in WS4, throughout the co‐design workshops we advocated for them by raising their views and needs identified from WS2 and including the findings in the final decision guide (Supporting Information: File S2).

#### 2.4.2 Co‐design workshops with family carers and professionals

**Figure 2:**
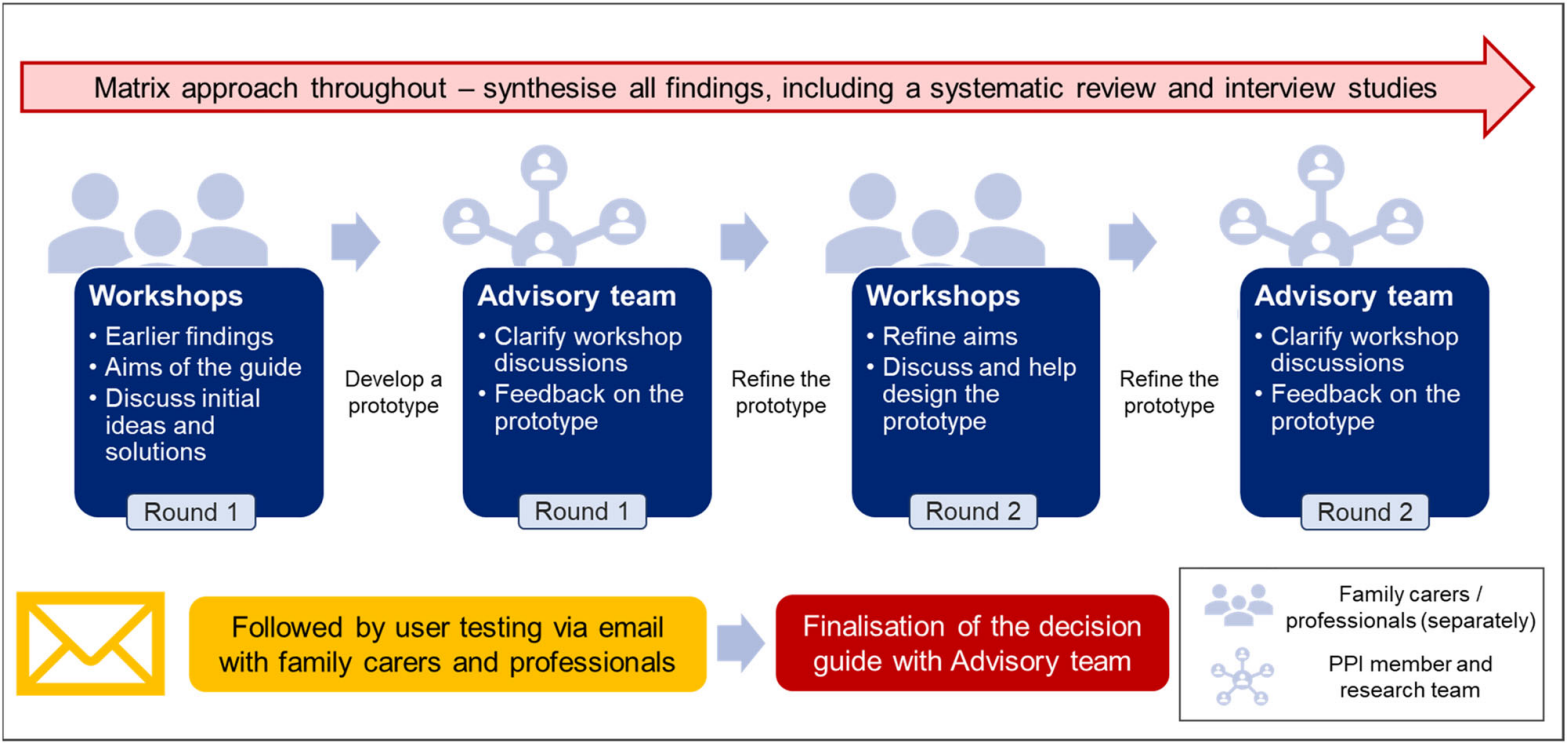
Overview of co‐design activities in Workstream 4

From May to September 2021, we held two co‐design workshops with family carers, two co‐design workshops with professionals and three research advisory team meetings. All workshops and meetings were online using Zoom software. Several versions of the prototype were discussed and iteratively refined through the co‐design workshops and research advisory team meetings (Supporting Information: File S1). We conducted user testing to get final feedback and finalize the decision guide (Supporting Information: File S2 for the final decision guide). A flowchart of co‐design workshop activities in this study is shown in Figure 2.

Although we acknowledged that a mixed group approach can unleash ideas that people might otherwise hold back and allows them to hear about other people's experiences,^36^ we conducted co‐design workshops with a homogenous group to minimize power imbalance among participants with different backgrounds,^37^ especially between family carers and professionals. However, we summarized and brought the ideas generated from one group to another to stimulate further discussions.

Timeframes and number of participants of co‐design workshops and research advisory team meetings are displayed in Table 2. The specific steps and activities of the co‐design process are described below.

**Table 2:** Timeframes and number of participants in workshops and research advisory team meetings

Online workshops or meetings	Month/year	No. of participants
Family carer workshop (round 1)	05/2021	8
Professional workshop (round 1)	06/2021	6
Advisory team meeting (round 1)	06/2021	7
Family carer workshop (round 2)	07/2021	6
Professional workshop (round 2)	08/2021	8
Advisory team meeting (round 2)	08/2021	7
User testing (individual, email)	08–09/2021	26
Advisory team meeting (round 3)	09/2021	7

##### Preworkshop preparation

We posted printed materials to participants in advance and asked them to use the materials on the day of the workshops (Supporting Information: File S3). They could record any ideas on these papers. This enhanced physical engagement and helped those with technology difficulties or who were less confident in speaking during the workshops. Participants were also able to understand the nature and expectations of our workshop activities and prepare some ideas to share, which is important in public engagement in research.^38^

##### Co‐design workshop procedures

All co‐design workshops lasted approximately 1.5 h and were facilitated by the first and second authors. We started by introducing workshop agreements mainly covering confidentiality and generating mutual respect. We emphasized some basic principles of a co‐design process and encouraged participants to express their ideas, listen to other people and ask questions whenever they wished (Supporting Information: File S3). We also navigated other methods of communication in the online software, for example, the use of the ‘Raise Hand’ and chat box functions to create a ‘safe’ and ‘brave’ space for participants to share their experiences and ideas and express disagreement.^36^

We used an online interactive board (Google Jamboard) to help facilitate and visualize discussion during our online workshops. However, we acknowledge that some people are not familiar with these technologies, and their use may hinder full engagement or make them feel uncomfortable. We, therefore, helped them type their views on the board and asked them to discuss them freely during the workshops. If participants wanted to express any ideas in writing, they could do so in the chat box function in Zoom.

##### Focus of discussions: First round of co‐design workshops

During the first workshops, we introduced the study background, described the overall study aims, presented findings from our WS2–3 interviews to participants and then started workshop activities. For both family carer and professional workshops, in the first rounds, we initially identified the difficulties of having conversations and decision‐making about eating and drinking for people with severe dementia during their hospital stay. We later focused on and creatively thought about possible solutions to the problems and initially discussed the design of our decision guide.

K. A., E. L. S. and N. D. then mapped the discussions in workshops and findings from our earlier workstreams into matrices (see below) as well as matters discussed in the first whole advisory team meeting to develop a prototype of the guide.

##### Focus of discussions: Second round of co‐design workshops

In the second round, discussions focused on the decision guide development. We recapped previous discussions and presented the prototypes (Supporting Information: File S1) to co‐design participants. A printed copy of the prototype was also sent to the participants in advance. This was to help participants comment on the physical version and visibility of the prototype, for example, font size, icons and colours. The co‐design groups then reflected on the prototype and generated ideas to improve it. They also discussed and refined the aims of the decision guide. After that, K. A., E. L. S. and N. D. developed a further iteration of the prototype based on the discussions and feedback from the co‐design groups with additional comments from the second advisory team meeting.

##### Postworkshop involvement

After each workshop, we created an electronic copy of the online discussion board and shared this with participants (Supporting Information: File S3). We encouraged participants to send us any additional thoughts on the workshop discussion, edits on the prototype and suggestions for the workshop process. Using a prepaid envelope, they could send us back the printed workshop materials with their notes and edits. We did not audio‐record workshops, but facilitators made detailed notes. We then compiled a report for research advisory team meetings. There was no formal analysis of the notes, but we scrutinized, summarized and aggregated the data into the matrices (Supporting Information: File S4).

#### 2.4.3 User testing

From August to September 2021, we conducted user testing by sending a near‐finalized prototype (Supporting Information: File S1) together with a feedback form (Supporting Information: File S5) to 40 people. The feedback form was adapted from a user manual for assessing the acceptability of a decision guide for osteoporosis treatments, as proposed by O'Connor and Cranney^39^ and is freely available to use. Those invited to this user testing were participants in our interview and workshop studies and people who had expressed interest but could not join the workshops.

The feedback form for user testing asked participants to rate the content of each section and comment on the amount of information, and the length of the nearly finalized version of the prototype. We also sought its perceived usability, that is whether the participants perceived the prototype would help start conversations and make decisions. The form also provided spaces for free‐text comments.

We offered participants the option of giving only qualitative feedback if preferred, either via emails, online meetings or telephone calls. Descriptive analysis was used to present the quantitative data, for example, frequency, proportion and percentage. There was no formal analysis of free‐text comments, but we included them in the matrices.

### 2.5 Data synthesis using a matrix approach

To develop the decision guide, we used a matrix to collate all findings from every workstream. The matrix was initially structured using a decision‐making model developed in the WS1—systematic review—to create the headings for rows. It helped prioritize problems and identify possible solutions to conversations and decision‐making about eating and drinking problems in acute hospital settings. It also informed the content, format and mode of delivery of the guide.

Data from every workstream were iteratively populated throughout the development process in WS4, and we subsequently modified key components of the matrix to enhance its clarity and thoroughness. Table 3 shows the key components of the matrix and indicates if data from the individual workstream was available for each component. A copy of the populated matrix is available in Supporting Information: File S4. Throughout the processes, we used the minimum criteria of the International Patient Decision Aid Standards (IPDASi) version 4.0 framework to inform the necessary components of our decision guide^40^ (Supporting Information: File S6).

**Table 3:** Matrix table for synthesizing and developing a decision guide

Key components	WS1 systematic review	WS2 PLWD interviews	WS3 family interviews	W3 professional interviews	WS4 workshops and research advisory team meetings
Common admitting conditions	NA	NA	x	x	x
Common eating/drinking problems at home or in care home	x	x	x	x	x
Common eating/drinking problems in hospitals	x	NA	x	x	x
Identify the problems and decisions	x	x	x	x	x
Initiate discussion or conversation	x	x	x	x	x
Exchange information: understanding disease and interventions	x	x	x	x	x
Exchange information: explaining disease and interventions	x	x	x	x	x
Acknowledge emotions of all involved	x	x	x	x	x
Clarify values of eating/drinking problems and interventions	x	x	x	x	x
Clarify values of approaching the *decisions*	x	x	x	x	x
Consideration of feasibility	x	x	x	x	x
Communicate preferred choices	x	x	x	x	x
Make a final decision and communicate the decision	x	x	x	x	x
Provide eating/drinking interventions	x	NA	x	x	x
Monitor and evaluate the support	x	x	x	x	x
Renegotiate the decision	x	x	x	x	x
Postdischarge education and support	NA	NA	x	x	x
Facilitators	x	NA	x	x	x
Barriers	x	NA	x	x	x
Decision guide: format	NA	x	x	x	See the last three rows below
Decision guide: techniques	NA	NA	NA	NA	See the last three rows below
Signpost additional information	NA	NA	NA	NA	x
Other components	x	NA	NA	NA	x
Decision guide: WS4 workshops and research advisory team meetings					
Aims and expected outcomes	x				
Preferred format	x				
Possible techniques to be used	x				

Abbreviations: NA, not applicable; PLWD, people living with dementia; WS, workstream.

## 3 FINDINGS

### 3.1 Participant characteristics

Most family carers and professional workshop participants continued their participation in our WS3 interview study. Many participants joined both co‐design workshops for each group. The co‐design workshop participant characteristics are shown in Table 4.

**Table 4:** Co‐design workshop participant characteristics

Participant characteristics	1st Family workshop (*n* = 8)	2nd Family workshop (*n* = 6)	1st Professional workshop (*n* = 6)	2nd Professional workshop (*n* = 8)
Participants from the interview study (WS3)	5	5	4	6
Age (years)				
Mean	46.5	46.5	42.2	44.8
Range	39–63	29–63	29–54	29–57
Sex (female: male)	5:3	4:2	6:0	7:1
Marital status				
Married or in a civil partnership	5	3	4	8
Single never married/in a civil partnership	2	3	1	0
Co‐habiting with partner	1	0	0	0
Divorced	0	0	1	0
Ethnicity				
English/Welsh/Scottish/Northern Irish/British	5	3	4	6
Asian/Asian British	1	0	2	2
Black Caribbean/Black British	0	1	0	0
Any other European White	2	2	0	0
Family carer participants				
Current caring situation				
Current carer	1	2	‐	‐
Bereaved or former carer	7	4	‐	‐
Relationship to the person with dementia				
Child	7	6	‐	‐
Grandchild	1	0	‐	‐
Hospital professional participants				
Current occupation				
Physician	‐	‐	1	2
Nurse	‐	‐	3	3
Speech and language therapist	‐	‐	2	2
Clinical psychologist	‐	‐	0	1
Years of working with people with dementia (year)				
Less than 1	‐	‐	0	0
1–5	‐	‐	2	2
5–10	‐	‐	1	0
More than 10	‐	‐	3	6

In user testing, 26 participants provided feedback and suggestions (response rate to invitation 65%). Of 26 participants, 22 participants used the feedback form. Four participants preferred to give qualitative feedback; two used emails, one chose an online individual meeting and another a telephone call. We did not collect demographic data, so the data are not presented here.

### 3.2 Key findings from workshops and research advisory team meetings

From the co‐design workshops and data synthesis using matrices, it seemed most participants were concerned about conversations or communications between family carers and professionals rather than decision‐making per se. For example, most would prefer risk feeding, in which people with dementia will be helped to eat as long as it does not cause them distress and to not use tube feeding. However, this can involve long and difficult conversations before reaching this decision. Through the co‐design process, the co‐design groups and research team developed the aims of the decision guide as being to support conversations and communication between family carers and professionals to reach informed shared decision‐making about eating and drinking problems in patients with severe dementia.

#### 3.2.1 Co‐design workshops with family carers and professionals

##### Common problems in conversation and decision‐making for eating and drinking and possible solutions

A voting exercise was used to identify and prioritize the problems and set some direction for group discussions about possible solutions. Separate lists of potential problems in the conversation and decision‐making for carer and professional workshops were predetermined and informed by the findings of WS1–3. We presented this list to participants and asked them to choose the problems that they perceived as most important. Using Zoom's voting function, they could choose multiple options from the list, and their choices were anonymous; however, we also explicitly asked them to raise any other issues they wished to discuss (Supporting Information: File S3).

From Table 5, family carers identified ‘finding the right staff to talk to’ (7 of 8 first workshop participants) as the most concerning problem, followed by difficulty ‘adapting to hospital rules and routines’ (5/8) and ‘addressing personal and cultural beliefs’ (5/8).

**Table 5:** Family carers and hospital professionals in the first round's identification of the most important points about conversations and decision‐making for eating and drinking^a^

Family carers	*N* = 8	Hospital professionals	*N* = 6
Finding the right staff	7	Know overall dementia prognosis	4
Hospital rules and routine	5	Explain treatment options	4
Personal and cultural beliefs	5	Hospital rules and routine	3
Available treatment options	3	Time pressure	3
Understanding about eating/drinking problems	3	Initiate conversation	3
Time pressure	2	Disagreement between people	3
Initiate conversation	2	Personal and cultural beliefs	2
Emotional difficulties	2	Explain eating/drinking problems	2
Disagreement between people	0	Confusing/underrecognized roles	2
		Emotional difficulties	1

^a^ Participants could choose more than one option (multiple choices).

In workshop discussions, family carers repeatedly mentioned difficulties in finding a constant point of contact and felt frustrated by having to say the same things to different professionals. They suggested having a live document or signs over beds that people could always access and update information about eating and drinking in the hospital. Family carers emphasized that empathy and mutual respect were important to help conversations run successfully. Therefore, professionals should offer sufficient time and be sensitive to their emotions and readiness for the discussion. This would then enhance the shared decision‐making processes.

From Table 5, professionals selected both ‘knowing the overall progression and prognosis of dementia’ and ‘explaining available treatment options’ as the most important issues during conversations and decision‐making (4 of 6 first workshop participants).

Professionals wanted to talk to the family member or carer who knew the person with dementia best to understand the overall progression of dementia and hear about existing eating and drinking problems and needs. This was in line with a person‐centred care approach. They also suggested using food and bowel charts as objective evidence to identify eating and drinking problems in hospitals and initiate conversations. These charts could help families to see eating and drinking changes and to build a more realistic understanding of the situation.

Professionals thought it was important that family members should talk to a member of the professional team who is familiar with and confident enough to discuss common eating and drinking problems of people with severe dementia. Otherwise, family members risk creating unrealistic expectations that may influence ensuing conversations. Many professionals wanted to inform families about some specific interventions provided in their hospitals, for example, finger foods, cultural menus and protected mealtimes. This was to allow families to come to hospitals and help communicate with the person and offer their preferred food and drinks. Professionals would also like to have electronic systems on which they could update the information they received from family members and share it with the wider health and social care providers.

All participants emphasized the cultural and personal meaning of eating and drinking, which should form part of the conversation. Language and communication difficulties could prevent people from explaining the situation or expressing their wishes. Professionals wanted to emphasize to family members that dementia was a terminal illness and to explore and validate their understanding and expectation about eating and drinking, especially at the end of life, while family carers wanted to know what the signs of end‐of‐life in dementia were, especially in terms of eating and drinking difficulties.

##### Content, format and mode of delivery of the decision guide

Throughout WS4, several versions of the prototype were presented and discussed in the co‐design workshops and research advisory team meetings (Supporting Information: File S1). Most participants agreed it could be a single version for both family carers and professionals to enhance a culture of trust and ensure that everyone would start the conversations from the same perspective. Therefore, both family carers and professionals stressed the decision guide should be sensitive to, but not underestimate, levels of understanding and knowledge of end‐users. For example, information should not make family carers feel patronized.

Although some participants suggested that the decision guide could be a booklet, most family carers and professionals agreed to keep it shorter (no more than two pages of A4) because it could be difficult for family carers or professionals to read long documents. The decision guide could be a conversation starter and signpost for people to further information resources elsewhere.

#### 3.2.2 Research advisory team meetings

Between the two rounds of workshops, the research advisory team helped interpret and expand the discussion from the workshops, and feedback on the prototype. For example, the team helped review the aims of the decision guide and suggested it could help professionals focus on engaging families in conversations, and vice versa, rather than mechanically going through all eating and drinking options. The decision guide might also benefit senior professionals for use in educational or training programmes for junior colleagues.

Food and bowel charts were thought useful subject areas to start the conversation and agree upon a care plan, but, at the end‐of‐life, professionals said they would not frequently record food, drink or whether the person has passed urine or had opened their bowels, so people could then stop using the charts. The team noted that signs over patients' beds were often left behind when patients moved to another bed or ward, so it would be more practical if the family kept the guide or information with them, ready to discuss with professionals.

### 3.3 User testing with family carers and professionals

#### 3.3.1 Content of each section of the prototype

**Figure 3:**
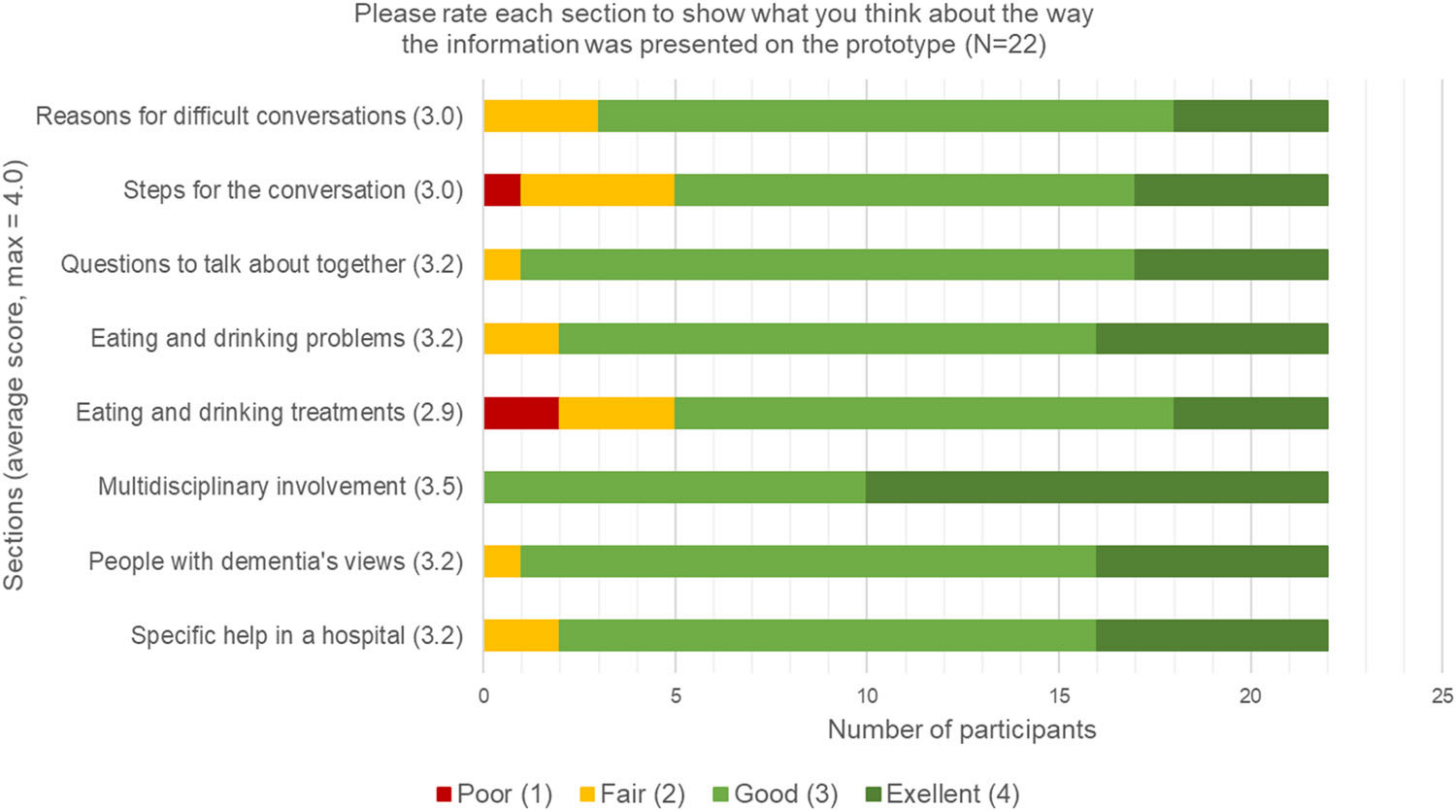
Average scores and rates of each section from the participants in the user testing

User testing was conducted on the nearly finalized version of the prototype (Supporting Information: File S1). As shown in Figure 3, participants could rate each section of the prototype according to the Likert scale of ‘poor, fair, good, and excellent’ (Supporting Information: File S5). To understand the overall rating, we converted these responses to the score of ‘1, 2, 3, and 4’, respectively, and then calculated the average scores of each section from all participants, as reflecting the Likert scale.

From the user testing, participants rated ‘good–excellent’ (average score 3.1–4.0) for most sections of the prototype. The introduction regarding a multidisciplinary approach was the most well‐received section. The eating and drinking treatment section was least favoured by participants, and one of the three sections was rated as ‘fair–good’ (average score 2.1–3.0), along with the ‘reasons for difficult conversations’ and the ‘steps of conversations’ section. This indicated these sections warranted further attention and reiterations.

#### 3.3.2 Overall presentation and perceived usability of the prototype

From Table 6, most participants thought the amount of information was ‘just right’ (68.2%) presented in ‘just right’ length of the prototype (86.4%). Every participant thought that the prototype could help people start a conversation about the eating and drinking problems of people with severe dementia in hospitals. Participants had mixed views about whether the information presented in the prototype was slanted particularly towards any eating and drinking treatments.

**Table 6:** Feedback on overall presentation and perceived usability of the prototype from the user testing

Feedback topics on the prototype	Family (*N* = 9)	Professional (*N* = 13)	Total (*N* = 22), *N* (%)
Participant type (total *N* = 26)^a^			
Participating in the interviews only	6	4	10 (38.5)
Participating in the workshops only	1	2	3 (11.5)
Both in interviews and workshops	3	6	9 (34.6)
People who expressed interest	0	4	4 (15.4)
Length of presentation			
Too long	0	3	3 (13.6)
Just right	9	10	19 (86.4)
Amount of information			
Too much information	2	5	7 (31.8)
Just right	7	8	15 (68.2)
Presentation slanted towards			
Use of tube feeding	0	1	1 (4.6)
Use of eating and drinking with accepted risks	0	7	7 (31.8)
Balanced	9	5	14 (63.6)
Do you think we included enough information to encourage a family member and hospital team get the conversation started?			
Yes	9	13	22 (100)
Would you have found this prototype useful when making your decision about eating and drinking for people with severe dementia in a hospital? (total *N* = 20)^b^			
Yes	7	10	17 (85.0)
No	0	3	3 (15.0)

^a^ Including four participants who provided only qualitative feedback.

^b^ Missing data from two former family carers who might have perceived this question was no longer relevant to them and so skipped the question.

Three professional participants (from 20 participants) thought information on the prototype might not be helpful in making their decisions. In free‐text comments, a professional participant explained that the information might not be useful for themself but could be for other professionals:

I personally wouldn't find this form useful for me … This is all information I am well aware of. BUT I do think the information is useful for doctors, however as a prompt to think of topics/issues they should be thinking about but often are not on a busy acute ward. I also think it does cover all of the key points for them.

Another two participants considered it was ‘too generic [and] each patient was different’ and that as ‘a general overview of the problem without necessarily helping [people] to reach a conclusion [and] as a prompt to initiate conversation it was helpful’. This was in line with the aims of the decision guide as defined by the co‐design groups to create a conversation facilitator for families and professionals rather than a self‐study document to reach a decision or conclusion by themselves. Similarly, professional workshop participants emphasized that the guide should not be prescriptive, and families and professionals should have a discussion to reach a personalized care plan.

#### 3.3.3 Qualitative feedback from free text comments, emails and one‐on‐one meeting

The participants' main concerns and suggestions concerned clarification of the purpose and of the end‐users of the guide at the beginning. Some suggested that every part was applicable and accessible for both family carers and professionals, in terms of comprehensibility and relevance. Although many participants thought the information was acceptable in its length and volume, they suggested that we condense the text and check the flow to enhance readability.

Some provided free‐text comments about the clarity and sensitivity of the language used. For example, some professional participants thought that the explanation about the disadvantages of tube feeding might be ‘too daunting’ to families, while family carers did not make any comments about this point. It was considered important to emphasize the multidisciplinary nature of involvement in eating and drinking for people with severe dementia in acute hospitals. It was thought worth explaining the roles of each professional in decision‐making processes as well.

Most participants mentioned they liked the guide because it contained sufficient, useful and concise information within a compact format. Overall, the sections had a clear layout using headings, blocks and colours; hence, they were easy to follow and could help get a conversation started.

### 3.4 Finalization of the decision guide

Based on the feedback and suggestions, we amended the prototype guide and cross‐checked with the matrix table to ensure consistency with findings from all workstreams. For example, when co‐design workshops suggested emphasizing the person‐centred and holistic approach, we then checked the sections (rows) of the matrix table across all workstreams (columns) that might be relevant to this, for example, the sections of ‘Acknowledge emotions of all involved’, ‘Clarify values of eating/drinking problems and interventions’ and ‘Clarify values of approaching the decisions’ (see Table 3 and Supporting Information: File S4).

In our last research advisory team meeting, the team helped interpret the findings from user testing and provided suggestions for the decision guide. Following this meeting, we continued refining and checking the guide met current evidence, guidelines and data synthesized from all workstreams. This was also checked against the IPDASi v4.0 criteria^40^ (Supporting Information: File S6). We worked with a professional designer to help redesign and convert the prototype into a decision guide. Following a final check with the research advisory team and workshop participants, we agreed that the decision guide could be finalized (Supporting Information: File S2).

### 3.5 Dissemination of the decision guide

From 1 December 2021, the final version of the decision guide (Supporting Information: File S2) was uploaded to the UCL Division of Psychiatry website and released via Twitter. We also emailed a copy of the decision guide with the link to the UCL Division of Psychiatry webpage to all participants, colleagues and relevant organizations. It was well‐received as people shared or recommended the guide to others. We heard that the decision guide has been circulated via email within local professional groups and uploaded to some organizations' electronic resources. One Admiral Nurse (dementia specialist) in an NHS trust offered to help with implementation.

### 3.6 Feedback on workshops and co‐design process

Throughout the co‐design process, we sought feedback and suggestions about workshops and the co‐design process from participants, including from the feedback form in user testing (Supporting Information: File S5). Many participants sent us emails after workshop sessions saying that they found the workshops engaging and thought‐provoking. In one of our first workshops, one participant suggested a queueing system for people to speak up because some participants might be not confident enough to do so. In the following workshops, we encouraged participants to use the ‘Raise Hand’ and ‘chat box’ functions, and this helped us to manage the queue and other people to build up discussions from their comments in the chat box more effectively.

Twelve workshop participants responded to the user testing process. In the feedback form, workshop participants mentioned the workshops were ‘well organised’, ‘easy to join and communicate’ and ‘naturally’ interactive, and it was nice to meet and exchange views with other people. It also made them feel that ‘[their] views mattered and counted which was great’ (former carer).

Some workshop participants suggested that the workshops could be ‘longer to cover all the points that were intended’ (current carer). Some were interested in having a workshop to bring persons with dementia, family carers and health professionals together to share ideas. Some thought it would be better to keep the same participants in all workshops because change could affect group dynamics and prevent discussion.

## 4 DISCUSSION

We applied systematic and transparent methods to develop a decision guide to be used by families and professionals covering nutrition and hydration for people with severe dementia in acute hospitals. We used various sources of evidence including a systematic review and interview and workshop studies, the data from which were synthesized. All processes interweaved principles of PPI in research and shared decision‐making in health and social care which have been encouraged in dementia and palliative care.^41^, ^42^ The study set a context within that families and professionals (the end‐users) can be heard and co‐design the guide with researchers, while the guide itself supported the shared decision‐making approach to dementia care decisions among those involved.

### 4.1 Practical points about a decision guide regarding the care for people with severe dementia in acute hospitals

#### 4.1.1 Aims and users of the decision guide

Consistent with our decision guide, the most common components of decision‐making guides appear to be tools to improve communication between persons with dementia, family and healthcare professionals.^43^ Our decision guide was co‐designed to be used in clinical discussions as a conversation facilitator for both families and professionals in a single document. Decision guides used in a discussion with other people involved in the decision‐making were also considered more beneficial than self‐guided tools.^44^

Decisions about eating and drinking interventions, either about risk feeding or artificial nutrition and hydration, involve personal values and cultural beliefs.^8^ We expect the guide would help, for example, people initiate conversations, families understand the working processes of the hospital team, and professionals recognize emotions and the personal meanings for the person with dementia and their family associated with eating and drinking. The decision guide provided sufficient and honest information to encourage further discussion. This is consistent with previous co‐designed research of a discussion tool about choices of care for people with dementia^35^ and decision guides in general.^19^

#### 4.1.2 Content presentation of the decision guide for dual users

In the co‐design process, family carers wanted honest and full information about all nutrition and hydration treatments in the guide. However, some professionals were not keen to include tube feeding because it is not recommended for people with severe dementia,^6^, ^42^ and so, if it was included, it could be seen as an alternative ‘option’. However, the core principles of a decision guide are to help a decision‐maker to make an ‘informed’ and ‘value‐sensitive’ decision.^19^ With the agreement of workshop participants and the research advisory team, we have included information about tube feeding in the guide and presented it in an honest way, based on current evidence.^6^, ^45^

Commenting on the presentation, family carers perceived the information as balanced and not distressing, but professional participants had very mixed opinions on this (see Table 6). It is worth noting that our decision guide was not a ‘patient decision guide’ that would be used by persons living with dementia, but by families and professionals to make decisions on behalf of the persons. Therefore, it was not entirely about the preferences of families and professionals, but rather it aimed to prompt everyone to consider important points and place the person with dementia at the centre of the discussion, in line with a person‐centred care approach.^46^

### 4.2 Key lessons from the co‐design process

Consistent with the literature,^36^ the co‐design process through workshop activities helped participants in this study feel heard by other participants who shared the same experiences, ideas and interests, and so they did not feel alone. The co‐design process helped them recognize other aspects of eating and drinking problems that they had never thought of, but could then prompt creative solutions or learning from other people's practice which they might consider using in the future.

Table 7 shows some lessons identified from this study that could be applied in future co‐design projects. Some of these lessons are discussed in more detail below.

**Table 7:** Lessons identified from this study for further co‐design research

Outline expectations about roles and workshop activities in advance
Start workshops with informal introductions (ice‐breaking)
Set workshop rules to create safe space and open discussion
Explain and agree workshop objectives and agendas
Consider any power hierarchy between different stakeholders
Be alert to possible exclusion from actual co‐design participation (tokenism)
Carefully plan and manage any lack of agreement among co‐design groups and between researchers
Observe and respond to person's emotions and needs
Observe and respond to group dynamic and relationships
Offer flexibility about time and location (more convenient for online workshops)
Consider varying ability and skills of each participant to take part in workshop activities
Consider suitability of workshop activities for intended participants and platform
Provide technological support, especially for online workshops
Offer variety of participating methods to maximize participation
Minimize any mismatch between the design of activities and the expected outcomes
Address any difficulty translating general conceptual ideas into practical solutions
Keep ongoing relationships and involvement with participants after the workshops
Provide sufficient time for each co‐design workshop
Consider time and financial constraints of entire research project

#### 4.2.1 Facilitate group dynamics and relationships

From the user testing feedback, a consistent group of participants across the co‐design process enabled us to sustain group dynamics and relationships and led to richer discussions. In a previous co‐design study, mutual understanding and trust within a co‐design group enabled a collaborative, compassionate and open mindset to collectively build on members' ideas and create solutions.^36^ However, making a commitment to participate in every session may be difficult for some participants, as can be seen, as some participants could not join the second workshop due to their unavailability. It was suggested participants are offered flexibility about their ability to participate.^47^ We explicitly informed participants that they were free to join all or any of the workshops, and we tried to accommodate the most convenient dates for most participants.

#### 4.2.2 Online platforms

All workshops in this study were online, and logistically this made the study an efficient use of time. It minimized the scheduling problems which can be a major problem of many face‐to‐face workshops.^48^, ^49^ However, the online platform hindered our use of certain types of co‐design exercises that could be useful, for example, patient journey or mind mapping, and paper prototyping and sketching. Some online interactive platforms can support these exercises, but these could have been too complex for some research participants. Training participating end‐users in research skills has been found helpful,^27^ which may include skills for these technologies. However, this was not feasible in this study due to time and financial constraints.

#### 4.2.3 Acknowledge and manage the lack of agreement

There was sometimes no agreement of ideas and preferences about the decision guide between researchers, family carers and professionals. This difference of opinion is common in co‐design studies and can compromise the study process and outputs and/or create feelings of tokenism and frustration and disappointment at the missed co‐design opportunity.^27^ However, it is the essence of co‐design research to facilitate participants in creatively thinking about ideas and agreeing, to some extent, with group conclusions, moving forward with specific solutions to the problems.

#### 4.2.4 Design of workshop activities: General concepts versus practical solutions

Workshop exercises can sometimes make it hard for participants to reach specific discussions and solutions within the workshop's pace and flow. For example, we found it easier for participants to instantly come up with the general aims of the guide and solutions to eating and drinking problems. It was more challenging when we specifically asked them how to frame the information in the guide, and this made one of our workshops rather quiet. It is important to carefully design and simplify exercises to ensure that they would not deter contributions to the discussions. This should be accompanied by sufficient time to respond using a variety of participatory methods, which was found to be effective in this study, for example, chat box and printed materials.

### 4.3 Strengths and limitations

This study used a systematic and transparent approach to co‐design a decision guide. It was based on our series of studies about the perspectives, experiences and needs of everyone involved, including people with mild dementia, family carers and professionals. Each study was done in sequence and informed subsequent studies. We involved PPI members and experts in palliative care and dementia care to feedback on study materials and interpret findings. We used the matrix table to synthesize all data and develop the guide, which allows for transparent reporting and was found beneficial in another co‐design study.^24^

All family workshop participants were recruited from JDR, and most of them continued their contribution to our interview study. These could be ‘super users’ who are constantly involved in research projects and might not represent the typical population of people with dementia and their carers.^47^ They could be a selected group of people who are keen on helping ‘design’ and participating in co‐design research.^29^ However, many family carers in our study were quite new to research involvement and had relatively mixed experiences and backgrounds. We triangulated family discussions with feedback from professional workshops and research advisory team meetings, including a PPI member, to help advocate for persons with dementia and other families.

### 4.4 Implications for policy, clinical practice and future research

Families and professionals can freely use the guide to prepare for or follow during such conversations and decision‐making (Supporting Information: File S2). However, we acknowledged possible barriers to implementing our decision guide in clinical practice, including indifference on the part of healthcare professionals and organizational inertia.^50^ Regarding participants' feedback, the guide could be used in clinical teaching to junior healthcare professionals or clinicians who are not fully confident in this area.

With adequate time and funding resources, further studies may strive to develop a process to involve people with dementia in co‐design workshops and to combine groups of participants and equalize their power, thereby fostering mutual and holistic understanding. A decision guide, for example, in a pictorial format may support people with dementia to indicate their preferences and further help family carers and professionals make decisions.

Future larger evaluation research may explore our decision guide's acceptability in real‐world settings. Standard tools may be used to measure, for example, decisional conflict, patient‐clinician communication, participation in decision‐making, decisional regrets and satisfaction with choices, as in previous studies.^19^, ^44^ A future development study may include an electronic format of a decision guide that would have more interactive and separate sections or layers of information and automatically direct users to information relevant to them.^35^

## 5 CONCLUSION

Through a rigorous co‐design process, we developed a decision guide to support conversations and decision‐making regarding nutrition and hydration for people with severe dementia in acute hospitals. From user testing, the decision guide was perceived to be useful in initiating conversations and making decisions. Co‐design workshop participants reported very positive experiences of being involved in our co‐design process. The final version of the decision guide was widely disseminated and is being used in clinical practice; however, it would be needed to be tested in future evaluation research.

## AUTHOR CONTRIBUTIONS

Kanthee Anantapong conceived and designed the study, recruited participants, acquired data from co‐design workshops, analysed and interpreted data, developed a prototype of the decision guide and wrote the manuscript. Andrea Bruun acquired data from co‐design workshops and contributed to the development of workshop materials and the interpretation of the data. Anne Walford, Christina H. Smith and Jill Manthorpe contributed to the development of workshop materials and the interpretation of the data. Elizabeth L. Sampson and Nathan Davies designed the study, supervised data collection and data analysis and contributed to the interpretation of the data. All authors reiterated the prototype of the decision guide, finalized the decision guide and revised and approved the final version of the manuscript to be published.

## CONFLICT OF INTEREST

The authors declare no conflict of interest.

## ETHICS STATEMENT

Ethical approval was granted by the Health Research Authority (Camden & Kings Cross Research Ethics Committee, REC reference: 20/LO/0049).

## Supporting information

Supporting information.

Supporting information.

Supporting information.

Supporting information.

Supporting information.

Supporting information.

## Data Availability

The data that supports the findings of this study are available in the supplementary material of this article.
