# Supplementary material for: Co‐design development of a decision guide on eating and drinking for people with severe dementia during acute hospital admissions
Source: Health Expect. 2023 Jan 17;26(2):613–29. doi: 10.1111/hex.13672 (PMC10010093; doi:10.1111/hex.13672)
Supplement: Supplementary file 1 — Supporting information. [file HEX-26--s004.pdf]

File S1: Versions of the decision guide prototype

After the first workshops with family carers and hospital professionals, this outline of a prototype was developed and presented in the first research advisory team meeting.

Guidance to conversation and decision-making around eating and drinking

for people with severe dementia during hospital admission

Carer page

Information you may want to know

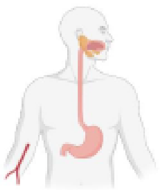

- Common eating problems + very basic physiology/anatomy of eating and drinking
- Eating and drinking options (oral feeding and ANH)
- Risk and benefits
- Hospital admission + acute illness/recovery vs dementia overall progression
- Common cultural belief (to encourage to speak out)
- Common misunderstanding (about eating/drinking at EoL, use of ANH)

Hospital staff who might be involved and you can talk to

- Physician/doctor (roles and what you can ask or tell about eating and drinking)
- Nurse
- Speech and language therapist
- Dietician (why split with SLTs)
- Palliative care team
- Healthcare assistants

Questions you may wish to ask the staff

- How PLWD is doing in the hospital?
- Why is PLWD not eating and drinking?
- What will be happening if I go with each option?
- What would make PLWD most comfortable?
- How much time do I have to make up my mind?
- How can we know if this is working?
- When will we think about this again?
- How can we talk again? (to have follow up conversation)

Notes to hospital team – what would you like them to know?

Page 1 of 2

DRAFT Nutri-Dem: H Guidance_v1.1_22.06.21

Hospital staff page

Common difficulties in having conversation/decision-making

- Difficult to find the right staff
- Consistency of information and communication
- Attitudes towards carers (and staff)
- Confidence in having conversation or confusing roles
- Safe space and enough time to discuss
- Feasibility in the hospital – hospital mealtimes and routine etc.
- Information retention 'stressful in hospital' – to have ongoing conversation or written information

Questions you may wish to ask the carers

- Baseline function, overall dementia progression
- Eating and drinking problems at home
- Check understanding about situation (dementia, acute illness and eating and drinking problems)
- What the carers have done, what would help eating and drinking in the hospital
- Support at home (Community support, GP, paid carers etc)
- PLWD's wishes, ACP
- Values or belief of PWLD and the carers (concerns and emotional difficulties)
- Can you show me how to give PLWD food and drink?
- Checking out the conversation – readiness to finish it (not one point – ongoing conversation, review the plan, ownership – reassuring – this is a plan – restating important points)

Information about your hospital support that you may wish to explain

- Protected mealtime policy in your hospital
- Hospital menu or cultural menu
- Personalised care plan 'forms' being used in your hospital - Food passport, This is me
- Adapted utensils
- Consultation – SLTs, dietician, palliative care
- Discharge support

Top tips about conversation/communication

- Empathy
- Don't patronise
- Address the language, communication difficulties
- What food means to PLWD and you (value) – what important to you
- Set of phrases and questions
- Food chart can help visualising the eating patterns and prognosis or dying process

Additional resources you may find useful (both for carers and hospital staff)

- Our guides with links
- Resource 2
- Resource 3

Very brief explanation about the guide development and research team and contact details/links to us

This prototype was presented to the second co-design workshop with family carers.

Guidance to conversation and decision-making around eating and drinking

for people with severe dementia during hospital admission

Instruction: You can use this guide to either prepare for conversations, follow during the conversations or recheck after the conversations. If preferred, family carers and health professionals can look into both sides.

Information you may want to know

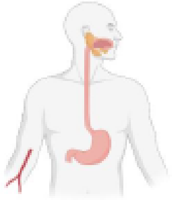

- Common eating problems + very basic physiology/anatomy of eating and drinking
- Eating and drinking options (oral feeding and artificial nutrition and hydration)
- Risk and benefits
- Hospital admission + acute illness/recovery vs dementia overall progression
- Common cultural belief (to encourage to speak out)
- Common misunderstanding (about eating/drinking at end of life, use of tube feeding and drips)
- Perspectives of people with dementia from earlier studies (respect their wishes, try offer food later, but also want to delegate decision to family – concerns about family's wellbeing)
- Try to condense the words down without losing any important point
- Sequential. Steps – real experience in the hospital – might not be linear
- Emphasise – engage, make use of the guide
- Staff advocate, saying that this is for you, might of help

Top tips for family carers about conversations

- Ask your relative or think of what they would really want for themselves would help the decision-making.
- Talking with someone who really takes care of food and drink for the person would be helpful for the staff.
- Having overall picture about how your relative has been doing at home would help staff think about care plan most appropriate to your relative.
- You may not need to make a decision at the time. Have a think at home and talk to other people (family, GPs) can be of help.
- Eating and drinking problems can involve multiple staff with different expertise, and the staff may also need some time to discuss a care plan with their team.

Questions you may wish to ask the staff

- How is my relative doing in the hospital?
- Why is my relative not eating and drinking?
- What will be happening if I go with each option?
- What would make my relative most comfortable?
- How much time do I have to make up my mind?
- How can we know if this is working?
- When will we think about this again?
- How can we talk again? (to have follow-up conversation)

Notes – what we have come up

....

....

....

....

Common difficulties about conversation and decision-making	Top tips for hospital staff
Many families find it difficult to find the right staff to talk to. Consistency of information and communication. Enough time to discuss.	Offer family the contact point or appointment to discuss
Attitudes towards carers (and staff)	Empathy, don't patronise, sensitive
Cultural differences (value)	What food means to person with dementia and family
Safe space	Ask if they want to discuss away from person with dementia
Confidence in having conversation or confusing roles	Food chart can help visualising eating patterns and prognosis, refer to senior or multidisciplinary team
Address the language, communication difficulties	Regular pauses, recheck understanding
Information retention 'stressful in hospital' –	Offer ongoing conversation and restate the important points or use written information
Feasibility in the hospital – hospital mealtimes and routine etc.	

Questions you may wish to ask the carers

- What do you understand about eating and drinking in people with dementia?
- Baseline function, overall dementia progression
- Eating and drinking problems at home
- What the carers have done? Can you show me how to give your relative food and drink?
- Support at home (Community support, GP, paid carers etc)
- The person with dementia's previous wishes, advance care planning
- Values or belief of the person with dementia and their carers (concerns and emotional difficulties)
- How do you feel about having this conversation?
- Checking out the conversation – readiness to finish it (not one point – ongoing conversation, review the plan, ownership – reassuring – this is a plan – restating important points)

Support specific to your hospital that you may wish to offer

- Protected mealtime policy in your hospital
- Hospital menu or cultural menu
- Personalised care plan 'forms' being used in your hospital - Food passport, This is me
- Consultation – roles of speech and language therapist, dietician, palliative care team
- Discharge support

More informational resources you may find useful (both for carers and hospital staff)

- Eating and drinking: Information for family and friends as dementia progresses towards the end of life (UCL resources) via https://www.ucl.ac.uk/psychiatry/sites/psychiatry/files/eating_and_drinking_final.pdf
- Supporting people who have eating and drinking difficulties (Royal College of Physicians) via https://www.rcplondon.ac.uk/projects/outputs/supporting-people-who-have-eating-and-drinking-difficulties
- Alzheimer's society website via https://www.alzheimers.org.uk/get-support/daily-living/eating-drinking

Very brief explanation about the guide development and research team and contact details/links

In the second workshop with hospital professionals, we tried to separate the decision guide into two versions: one for family carers and one for hospital professionals. This was to test whether separate versions were needed. However, the co-design workshop suggested to have one version for all to enhance trust among the users.

Family carer page

Guidance to conversation and decision-making about eating and drinking for people with severe dementia during hospital admission

Instruction: You can use this guide to either prepare for conversations, follow during the conversations, or reread it after the conversations. If preferred, family carers and health professionals can look into both versions.

Eating and drinking: the problems resulting from dementia

Normal eating and drinking process is hugely involved brain functions on motivation and feelings (hunger, thirst, satiety, pleasure), recognition of food and utensil, and movements of muscles (in mouth and throat). Dementia can affect these functions and cause the person to eat/drink less, eat too much or eat odd food. They can find it difficult to focus on eating/drinking at a time or maintain regular eating routine. Swallowing can become more problematic later, and this can cause choking (food going into the windpipe). Elimination problems like constipation and incontinence can also cause distress and impact on eating and drinking. Due to dementia progression, levels of hunger and required energy can be reduced.

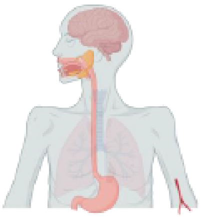

Acute medical illnesses like stroke, infection and falls can cause or worsen eating and drinking problems. Hospital environment can be confusing for people with dementia, especially for those with eye-sight or hearing deficits. The acute illnesses can sometimes be treated, and the eating problems can be improved. But the overall dementia progression of the person would not be changed or even rapidly declined in some cases, resulting in further eating problems.

Common eating and drinking options

Risk feeding – offer food when requested by the person.	Tube feeding – give liquidised food by a tube inserted via nose or abdominal wall into stomach.	Drips – deliver fluids by a tube inserted via skin into blood vessels.
Pros: support pleasure of eating/ drinking by mouth; led by the person's wishes	Pros: controllable food amounts	Pros: rapid and temporary re-hydration; medium to give drugs for treating acute illnesses.
Cons: risks of chocking, but can be managed to acceptable levels.	Cons: don't improve nutrition, prevent choking or prolong life; can cause great distress.	Cons: can cause distress to the person; easily be pulled out.

Who can be involved in the care for eating and drinking

- Speech and language therapist or SLT assess communication and swallowing capacity; advise on safe eating and drinking
- Dietician: assess and advise on nutritional requirement; prescribe supplements
- Healthcare assistants: support eating and drinking at bedside; monitor/record eating and drinking
- Palliative care team: assess and plan for the care at the later stages to promote comfort of the person with dementia
- Nurse
- Physician

Family can talk to physician and nurses in charge, and the allied staff to help decision-making.

When being asked about future eating and drinking problems, **people with mild dementia** often want hospital staff to help their family making the decisions. They concern about their family's wellbeing. But they still want family and staff to respect their wishes and look at what they say or do at a time. Food can be offered later if they refuse. They mostly don't like the ideas of tube feeding and want to be most comfortable, especially at the later stages or end of life.

Food and drink generally have unique meanings to the person living with dementia and their family. Food and drink can be the ways people express love and care for their loved ones, which is very important in some cultures. Eating and drinking can also be social activities that people share time, enjoyment and memories.

More informational resources you may find useful

- Eating and drinking: Information for family and friends as dementia progresses towards the end of life (UCL resources) via https://www.ucl.ac.uk/psychiatry/sites/psychiatry/files/eating_and_drinking_final.pdf
- Supporting people who have eating and drinking difficulties (Royal College of Physicians) via https://www.rcplondon.ac.uk/projects/outputs/supporting-people-who-have-eating-and-drinking-difficulties
- Alzheimer's society website via https://www.alzheimers.org.uk/get-support/daily-living/eating-drinking

This guide was developed and co-designed using evidence from interviews and workshops with older people with mild dementia, family carers and hospital staff. Research team consists of old age psychiatrists, psychologists, speech and language therapists, social care researchers, and conversational analysts.

Guide: eating & drinking decisions for people with dementia in hospitals_family_DRAFT_V2.1_27.07.21 Page 1 of 2

Common difficulties you may find when having conversation in a hospital

First day(s)

Family may receive uncertain information about care plan about acute illnesses, including eating and drinking problems. Hospital team may need time to know the exact causes of the illness and hold eating and drinking by mouth. Temporary support may be used such as drips.

Over the time

Many hospital staff can be involved in eating and drinking care plan and come to talk to the person and family. Staff can constantly change over the shifts. This can make family feel worried to repeat things to different staff. Some family can be unfamiliar with the roles of some experts, for example, speech and language therapists and palliative care team. Family may feel uncomfortable discussing care plan in front of the person. Family may not be allowed to stay for long in the hospital and do not receive regular updates. Family may also feel unsure when and how they should contact the hospital team when the family are not in the hospital.

Discharge

Before getting back home, family usually receive insufficient information, especially about how eating and drinking problems can progress in the future and what they can do about that.

Advices for family carers about the conversations

The person with severe dementia can sometimes express what they would like to eat or drink at a time. This can change from time to time. So, we can observe and regularly check with them.

Eating and drinking decisions depend mainly on the stages of dementia and prognosis of acute illnesses. Talking to someone who know best about how the person has been doing at home (or care home) can help staff make decisions and draw the goals of care and overall care plan.

You may not need to make a decision right at the conversation. You can go back home, find more information yourself and talk to other people (family members, friends, GPs etc).

Some hospitals may allow family bringing food in and helping with eating and drinking at a certain time of the day. Staff are keen to learn from family on how to give food and drink to the person.

Questions you may wish to ask hospital staff

- How is my relative doing in the hospital?
- Are they having any eating and drinking problems?
- What could be the causes of eating and drinking problems?
- Are the problems related to their acute illness?
- Are the problems temporary or will they be progressing?
- What are all the possible support or treatment?
- What will come next for each option? Risks and benefits?
- What would make my relative most comfortable?
- How much time do I have to make up my mind?
- When and how can we know if this is working?
- When and how will we talk about this again?

To make eating and drinking decisions, family may fear of not doing enough for their loved one. Talk to hospital staff and explain about your points and feelings can help the staff understand your views, emotions and needs.

Goals of care in the later stages should aim to promote comfort and dignity of the person living with dementia, not to provide complete nutrition.

Notes – what have we come up so far and what do you wish to speak to the staff next time?

Guidance to conversation and decision-making about eating and drinking for people with severe dementia during hospital admission

Instruction: You can use this guide to either prepare for conversations, follow during the conversations, or reread it after the conversations. If preferred, family carers and health professionals can look into both versions.

Eating and drinking: the problems resulting from dementia

Normal eating and drinking process is hugely involved brain functions on motivation and feelings (hunger, thirst, satiety, pleasure), recognition of food and utensil, and movements of muscles (in mouth and throat). Dementia can affect these functions and cause the person to eat/drink less, eat too much or eat odd food. They can find it difficult to focus on eating/drinking at a time or maintain regular eating routine. Swallowing can become more problematic later, and this can cause choking (food going into the windpipe). Elimination problems like constipation and incontinence can also cause distress and impact on eating and drinking. Due to dementia progression, levels of hunger and required energy can be reduced.

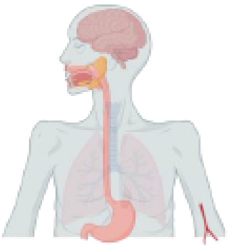

Acute medical illnesses like stroke, infection and falls can cause or worsen eating and drinking problems. Hospital environment can be confusing for people with dementia, especially for those with eye-sight or hearing deficits. The acute illnesses can sometimes be treated, and the eating problems can be improved. But the overall dementia progression of the person would not be changed or even rapidly declined in some cases, resulting in further eating problems.

Common eating and drinking options

Risk feeding – offer food when requested by the person.
Pros: support pleasure of eating/ drinking by mouth; led by the person's wishes
Cons: risks of chocking, but can be managed to acceptable levels.

Tube feeding – give liquidised food by a tube inserted via nose or abdominal wall into stomach.
Pros: controllable food amounts
Cons: don't improve nutrition, prevent choking or prolong life; can cause great distress.

Drips – deliver fluids by a tube inserted via skin into blood vessels.
Pros: rapid and temporary re-hydration; medium to give drugs for treating acute illnesses.
Cons: can cause distress to the person; easily be pulled out.

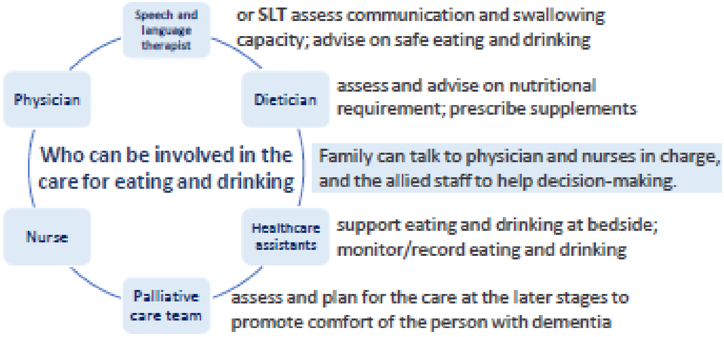

Speech and language therapist or SLT assess communication and swallowing capacity; advise on safe eating and drinking

Dietician: assess and advise on nutritional requirement; prescribe supplements

Family can talk to physician and nurses in charge, and the allied staff to help decision-making.

Healthcare assistants: support eating and drinking at bedside; monitor/record eating and drinking

Palliative care team: assess and plan for the care at the later stages to promote comfort of the person with dementia

When being asked about future eating and drinking problems, **people with mild dementia** often want hospital staff to help their family making the decisions. They concern about their family's wellbeing. But they still want family and staff to respect their wishes and look at what they say or do at a time. Food can be offered later if they refuse. They mostly don't like the ideas of tube feeding and want to be most comfortable, especially at the later stages or end of life.

Food and drink generally have unique meanings to the person living with dementia and their family. Food and drink can be the ways people express love and care for their loved ones, which is very important in some cultures. Eating and drinking can also be social activities that people share time, enjoyment and memories.

More informational resources you may find useful

- Eating and drinking: Information for family and friends as dementia progresses towards the end of life (UCL resources) via https://www.ucl.ac.uk/psychiatry/sites/psychiatry/files/eating_and_drinking_final.pdf
- Supporting people who have eating and drinking difficulties (Royal College of Physicians) via https://www.rcplondon.ac.uk/projects/outputs/supporting-people-who-have-eating-and-drinking-difficulties
- Alzheimer's society website via https://www.alzheimers.org.uk/get-support/daily-living/eating-drinking

This guide was developed and co-designed using evidence from interviews and workshops with older people with mild dementia, family carers and hospital staff. Research team consists of old age psychiatrists, psychologists, speech and language therapists, social care researchers, and conversational analysts.

Conversation in a hospital: common difficulties and possible tactics

Difficult to grasp the overall picture of dementia progression and set overall goals of care	Seek and talk to family or carer who know best about how the person has been doing at home (or care home)
Difficult to provide consistent point of contact due to changing staff over the shifts and off-hours	Offer family the contact details, availability, or appointment for formal discussion; notes over weekends
Conflicting attitudes among family carers and hospital staff to each other	Patient-centred always a priority; consider family as a team; empathic listener; aware of non-verbal
Uncertain to give definite causes and prognosis of acute illnesses and eating and drinking problems	Acknowledge the limitation but also giving a clear and honest plan of further investigation and care
Unclear roles and responsibility, then difficult to get overall picture of eating and drinking problems	Regularly updated food chart can help visualise eating patterns and prognosis and be used in a conversation
Feel lacked confident to have conversations and make decisions	Consult or debrief with a more senior staff; arrange a multidisciplinary team or best interest meeting
Feel unprepared to address the differences of cultural beliefs and values of a person with dementia and family	Impossible to understand every culture, so learn from the person and family about what food means to them
Communication difficulties including language barriers and limited time	Provide regular pauses, recheck understanding; use simple language and offer a formal translator if available
Stressful hospital situation can impact the person and family's ability to understand and retain information	Offer follow-up conversations and restate the important points; or use written information
Limited space to have conversation, many don't want to do it at bedside in front of the person with dementia	Ask if they want to discuss away from the person with dementia; most families prefer a private and quiet space
Signs or information not being passed when the patients are transferred to different wards/bays	Regularly check the signs over the bed and update information when having something new
Concerns about practicality and feasibility in the hospital like hospital mealtimes and routine	Be flexible and ask the family if they have any ideas or tips to help eating and drinking in the hospital

Support specific to your hospital that you may wish to offer

There could be some support available in your hospital (hugely varied across different settings), but some support might not be recognised or regularly used. For example, they may allow family helping with eating and drinking during 'protected mealtimes'. Catering services may offer cultural food and drink in their menu. Some forms like 'Food Passport' or 'This is Me' can help personalise eating and drinking care plan. Consultations with other expertise like speech and language therapists, dieticians and palliative care team can help with the conversation and decision. Links with community team like GPs can help develop long-term care plan. You may try to be familiarised with the support and offer them to the person and family.

Questions you may wish to ask family or carers

- How has your relative been doing at home (or care home) in general?
- What do you understand about dementia and how it progresses?
- What do you understand about eating and drinking in people with dementia?
- Has your relative had any eating and drinking problems at home?
- What have you tried to help with the problems?
- Have you received any support from community? (community services, GP)
- Would you like to show us how to give or offer your relative food and drink?
- Does your relative need eye-glasses or hearing aids? Any false teeth?
- Has your relative mentioned anything about their wishes or preferences on eating and drinking? Maybe from the experiences of caring for someone else.
- What could food and drink mean to you and your relative?
- What would other people in your family think about providing or stopping food and drink to someone?
- How do you feel about having the conversation about eating and drinking?
- Is there any part of our conversations today you find it not making sense?
- Do you have any questions before we leave today? Are you ready to finish?

Family can be worried about what would happen after the conversation and if they could meet the team again. You may wish to reassure that there is a care plan, and give them initial timelines of treatments and future discussion(s).

Goals of care in the later stages should aim to promote comfort and dignity of the person living with dementia, not to provide complete nutrition.

The prototype was presented at the second research advisory team meeting. This version was to be used by both family carers and hospital professionals. After a few minor amendments, this version of the prototype was sent to participants in the user testing together with the feedback form.

UCL

For both family and hospital staff

Talking about eating and drinking for people with severe dementia during hospital stays

Why can the conversation be difficult in a hospital?

Uncertainty about causes and treatments

Emotionally involved; cultural and personal meaning of food and drink

Limited time and staffing resources in the hospital

Steps of the conversation and decision-making – things to consider and possible tactics

**Recognise and start conversation**	• Check or inform if the person has problems with eating and drinking at home<br>• Ask nurses and healthcare assistants about the person's eating and drinking<br>• Share food and bowel charts to identify the problems and prompt conversations
**Exchange information** (more in the next page)	• Overall progression of dementia: how the person has been doing at home in general<br>• Possible causes of and treatments for eating and drinking problems in hospital<br>• Tips from family to help the person eat and drink – share them with staff<br>• Finding the exact cause can take time and involve different members of staff
**Recognise emotions**	• Family frustration about uncertain information and repeating things to different staff<br>• Staff unsure about how much family wants to know and what to say when<br>• Be sensitive and aware of communication difficulties and language barriers<br>• Provide private and quiet space (some prefer to discuss this away from the patient)<br>• Take regular pauses and rechecks, restate important points, outline overall care plan
**Talk about values and beliefs**	• Discuss previous preferences and wishes of the person with dementia<br>• Hard to understand every family and their culture – ask what food means<br>• Talk about overall goals of care for the person and what actually can be done<br>• Different views or uncertainty may be helped by multidisciplinary talking<br>• Be clear about the law on best interests decision-making
**Make and revisit decisions**	• In hospital, many eating and drinking are medical decisions for the medical team, but inputs from carers can help the team work out what is the best thing to do<br>• Family needs time to think and talk to others including GPs when making decisions<br>• Discuss timelines of the chosen treatment: how to monitor if things work or change<br>• Use notes, provide contact details and availability for follow-up conversations<br>• Discuss and ask for discharge notes and care plan at home (or care home)

? Questions you can think of and may wish to ask (or explain)

- What does the family know so far about dementia and its progression?
- How does the person eat and drink at home?
- Would family like to show staff how to offer the person food and drink?
- What could be the causes of eating and drinking problems in the hospital? Are the problems temporary?
- What are the ways to help? What would happen next for each way?
- Has the person mentioned anything about their wishes or preferences on eating and drinking? Maybe the experiences of caring for someone else
- Is there any part of our conversation today that did not make sense or was more difficult to follow? Are you ready to finish?
- When and how can we know if the chosen treatment is working? When and how will we talk about this again?

To make eating and drinking decisions, family may fear not doing enough for their relative. Explaining your points and feelings can help the hospital staff understand your views, emotions and needs.

Goals of care in the later stages should promote the comfort and dignity of the person living with dementia.

Guide: eating & drinking decisions for people with dementia in hospitals_DRAFT_V3.4_23.08.21 Page 1 of 2

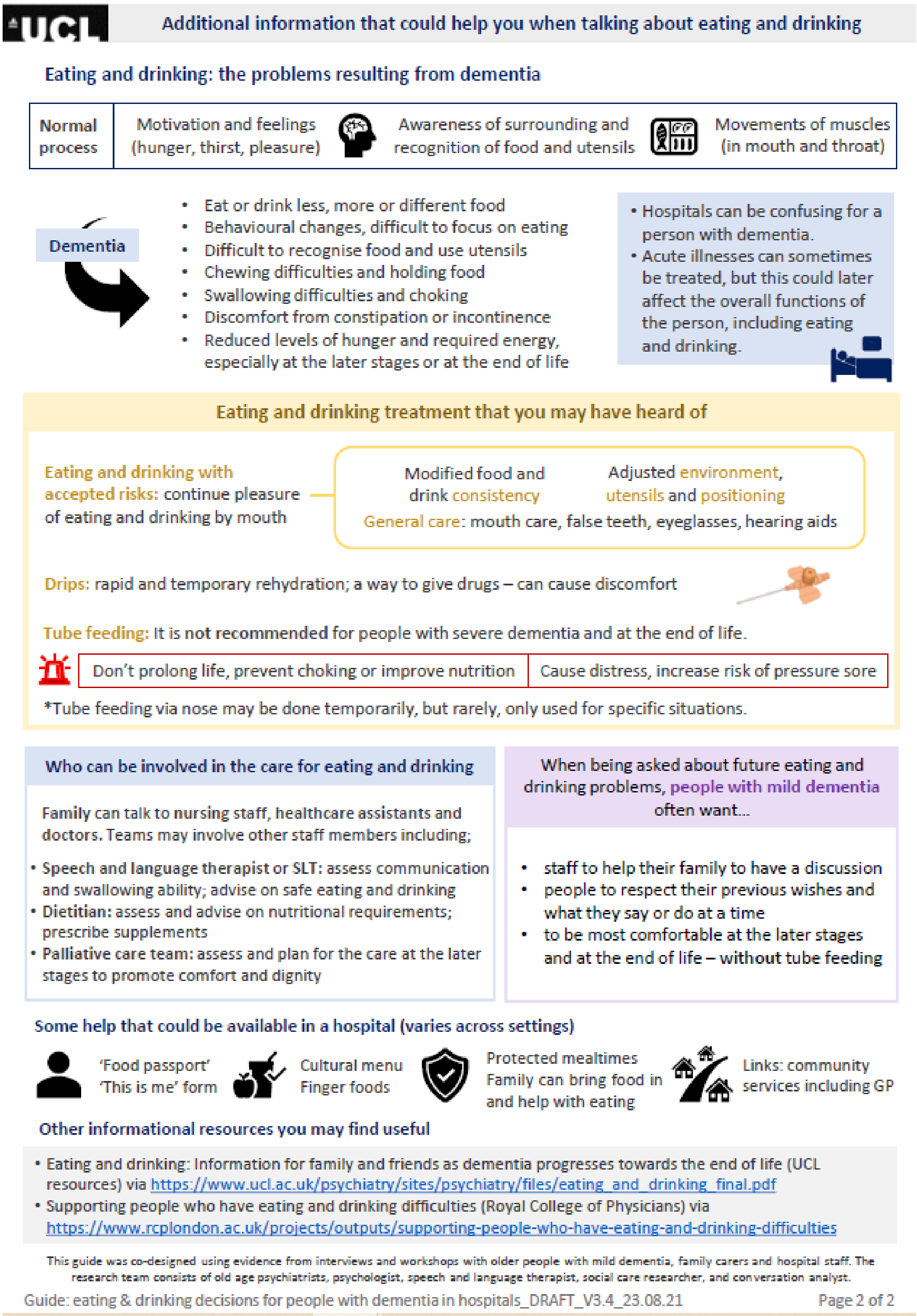
UCL Additional information that could help you when talking about eating and drinking

Eating and drinking: the problems resulting from dementia

Normal process	Motivation and feelings (hunger, thirst, pleasure)	Awareness of surrounding and recognition of food and utensils	Movements of muscles (in mouth and throat)

Dementia

- Eat or drink less, more or different food
- Behavioural changes, difficult to focus on eating
- Difficult to recognise food and use utensils
- Chewing difficulties and holding food
- Swallowing difficulties and choking
- Discomfort from constipation or incontinence
- Reduced levels of hunger and required energy, especially at the later stages or at the end of life

- Hospitals can be confusing for a person with dementia.
- Acute illnesses can sometimes be treated, but this could later affect the overall functions of the person, including eating and drinking.

Eating and drinking treatment that you may have heard of

Eating and drinking with accepted risks: continue pleasure of eating and drinking by mouth

Modified food and drink consistency

Adjusted environment, utensils and positioning

General care: mouth care, false teeth, eyeglasses, hearing aids

Drips: rapid and temporary rehydration; a way to give drugs – can cause discomfort

Tube feeding: It is not recommended for people with severe dementia and at the end of life.

Don't prolong life, prevent choking or improve nutrition	Cause distress, increase risk of pressure sore

*Tube feeding via nose may be done temporarily, but rarely, only used for specific situations.

Who can be involved in the care for eating and drinking

Family can talk to nursing staff, healthcare assistants and doctors. Teams may involve other staff members including;

- Speech and language therapist or SLT: assess communication and swallowing ability; advise on safe eating and drinking
- Dietitian: assess and advise on nutritional requirements; prescribe supplements
- Palliative care team: assess and plan for the care at the later stages to promote comfort and dignity

When being asked about future eating and drinking problems, people with mild dementia often want…

- staff to help their family to have a discussion
- people to respect their previous wishes and what they say or do at a time
- to be most comfortable at the later stages and at the end of life – without tube feeding

Some help that could be available in a hospital (varies across settings)

'Food passport' 'This is me' form

Cultural menu Finger foods

Protected mealtimes Family can bring food in and help with eating

Links: community services including GP

Other informational resources you may find useful

- Eating and drinking: Information for family and friends as dementia progresses towards the end of life (UCL resources) via https://www.ucl.ac.uk/psychiatry/sites/psychiatry/files/eating_and_drinking_final.pdf
- Supporting people who have eating and drinking difficulties (Royal College of Physicians) via https://www.rcplondon.ac.uk/projects/outputs/supporting-people-who-have-eating-and-drinking-difficulties

This guide was co-designed using evidence from interviews and workshops with older people with mild dementia, family carers and hospital staff. The research team consists of old age psychiatrists, psychologist, speech and language therapist, social care researcher, and conversation analyst.

Guide: eating & drinking decisions for people with dementia in hospitals_DRAFT_V3.4_23.08.21 Page 2 of 2

After user testing and the last research advisory team meeting, we continued working with a professional designer to iteratively amend the prototype and transform it into the final version of the decision guide.
