# Supplementary material for: Co‐design development of a decision guide on eating and drinking for people with severe dementia during acute hospital admissions
Source: Health Expect. 2023 Jan 17;26(2):613–29. doi: 10.1111/hex.13672 (PMC10010093; doi:10.1111/hex.13672)
Supplement: Supplementary file 2 — Supporting information. [file HEX-26--s005.pdf]

File S2: Final version of the decision guide

The final version of our decision guide is attached in this supplementary file. The final version of the decision guide is protected by copyright but is freely available to share and use in practice and research, provided the reference is cited in any research publications or reports. Please visit the links below.

Original URL:

https://www.ucl.ac.uk/psychiatry/sites/psychiatry/files/eating_drinking_hospital_dementia.pdf

Short URL for sharing: https://tinyurl.com/uclEatingDrinkingHospital

Suggested citation: Please cite this published article

Correspondence: Dr Kanthee Anantapong

Email: kanthee.a@email.psu.ac.th ; kanthee.anantapong.18@ucl.ac.uk

ResearchGate: https://www.researchgate.net/profile/Kanthee-Anantapong

Twitter: @KantheeMD

UCL

Talking about eating and drinking for people with severe dementia during hospital stays

Eating and drinking matters to everyone. It can be **hard** for family and hospital staff to talk about eating and drinking problems. People can be unsure about causes, outcomes, and treatments.

It is always good to talk to and observe the person. Sometimes people with severe dementia can say or do things to express what they would like to do about eating and drinking.

Conversation & decision-making steps

1. Recognise problems and start the conversation

Eating and drinking patterns
Existing difficulties at home
Changes while in hospitals

Use food, fluid and bowel charts to spot problems and prompt conversations

How does the person eat and drink at home and in hospital?

2. Share information

- Progression of dementia
- General needs: glasses, hearing aids, dentures
- Eating and drinking problems

Finding the cause
Can take time and involve different members of staff

Action
Tips from family to help the person eat and drink

- What do you know about the person's dementia?
- What is causing eating and drinking problems?
- Are the problems temporary?
- What are the best ways to help?
- Would family like to show staff how to help their relative eat and drink?

3. Recognise emotions

- Frustration about uncertainty and repeating things to different people
- Unsure about how much information is needed, what to say, and when
- Communication difficulties and language barriers

Try to find private space

Recheck
Use notes, regularly pause and repeat important points

Is there any part of our conversation today that did not make sense or was more difficult to follow?

4. Talk about values and beliefs

Patient at the centre
Previous preferences and wishes

Clinical team discussion
Can help sort out different views or uncertainty

- Discuss overall care for the person
- Best interests decision-making

- Has the person mentioned anything about their wishes or preferences for eating and drinking?
- What does food mean to the person and family?

5. Make decisions and follow-up conversations

- Many eating and drinking decisions are made by the clinical team, but advice from family will help the team work out the best thing to do
- Enough time for everyone to think and talk to others
- Share contact details and availability for follow-up conversations

Chosen treatments
Timelines and how to monitor

Advance care plan
Care plan that makes a record of wishes and decisions

- Have you managed to say what you wanted to say?
- When and how can we know if the chosen treatment is working?
- When and how will we talk about this again?

When making eating and drinking decisions, people may fear that the person may starve. Discussing concerns and feelings can help everyone understand each other's personal views, emotions and needs.

The goals of care in the later stages of dementia should promote comfort and dignity for the person living with dementia.

Additional information

Eating and drinking difficulties that may arise for a person living with dementia

Dementia can affect...

- Motivation and feelings (appetite, hunger, thirst, pleasure)
- Recognition of surroundings, food and utensils
- Movement of muscles (in mouth and throat)

- Eat or drink less
- Eat more or different food
- Behaviour changes, difficult to focus on eating
- Difficult to recognise food and use cutlery
- Difficulty chewing and holding food in the mouth
- Swallowing difficulties, coughing and choking
- Reduced levels of hunger and calorie needs, especially at the later stages or near the end of life

Eating and drinking treatments that you may have heard of

Eating and drinking with accepted/known risk:
Continued enjoyment of eating and drinking by mouth

- Modified food and drink **consistency**
- Adjustments to **environment**, **utensils** used and **positioning of person**
- **A clean mouth** (teeth and tongue) is pleasant and safe

Drips:
Rapid and temporary rehydration. A way to give drugs and fluids but can cause discomfort.

Tube feeding:
Is **not recommended** for people with severe dementia. It does not prevent choking, improve nutrition, nor prolong life. It can cause the person distress and increase risks of pressure sores. Temporary tube feeding via the nose may be useful only in very specific situations and needs clinical team discussion.

Who can be involved?

Family can talk to **nurses**, **healthcare assistants** and **doctors**. Teams may include:

- **Speech and language therapist** or **SLT**: assess communication and swallowing ability; advise on safe eating and drinking
- **Dietitian**: assess and advise on nutritional requirements; prescribe supplements
- **Palliative care team**: assess and plan for care in the later stages of dementia to promote comfort and dignity

When asked about future eating and drinking problems, **people with mild dementia** often want…

- Staff to help their family have a discussion
- People to respect their previous wishes and preferences
- To be as comfortable as possible at the later stages/end of life – without tube feeding

Some help that could be available in a hospital
(varies across different hospitals)

- 'Food passport'
  'This is me' form
- Cultural menu
  Finger foods
- Protected mealtimes
  Family can bring food in and help with eating

Other resources you may find useful

- Eating and drinking as dementia progresses towards the end of life via https://tinyurl.com/uclEatingAndDrinking

This guide was co-designed using the latest evidence, guidelines and interviews/workshops with people with mild dementia, family carers and hospital staff. The research team consists of old age psychiatrists, psychologist, speech and language therapist, social care researcher, and conversation analyst.

Kanthee Anantapong 2021 ©, Twitter: @KantheeMD
Liz Sampson, Nathan Davies, Jill Manthorpe, Christina Smith, Andrea Bruun, Anne Walford
Design: Rasmus Klaustrup
Version 1.0 November 2021
