# Supplementary material for: Co‐design development of a decision guide on eating and drinking for people with severe dementia during acute hospital admissions
Source: Health Expect. 2023 Jan 17;26(2):613–29. doi: 10.1111/hex.13672 (PMC10010093; doi:10.1111/hex.13672)
Supplement: Supplementary file 3 — Supporting information. [file HEX-26--s001.pdf]

File S3: Examples of online workboard and hand-outs

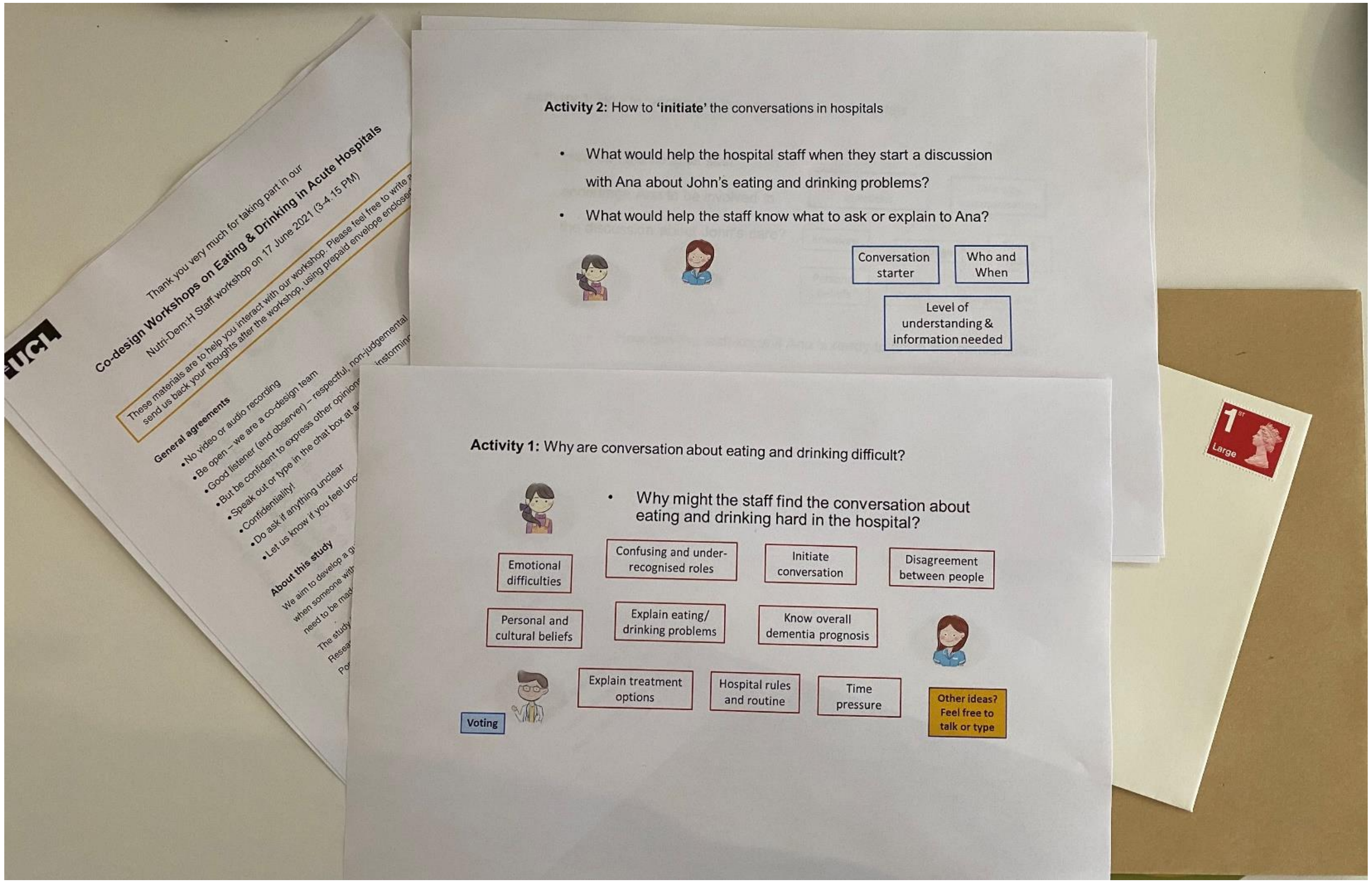

General agreements

- No video or audio recording
- Be open – we are a co-design team
- Good listener (and observer) – respectful, non-judgemental
- But be confident to express other opinions (brainstorming)
- Speak out or type in the chat box at anytime (can use 'Raise Hand')
- Confidentiality!
- Do ask if anything unclear
- Let us know if you feel uncomfortable (use the private chat)

The workshop materials are to facilitate our interactions during workshop activities.

Please feel free to note your thoughts on them. We also sent you an initial draft of our guide – please freely make changes, remove or add anything to the guide as you wish.

You can send us back your thoughts and edits using the prepaid envelope enclosed in the letter.

Google Jamboard

Examples from the first workshop with family carers

Activity 1: Why are conversation about eating and drinking difficult?

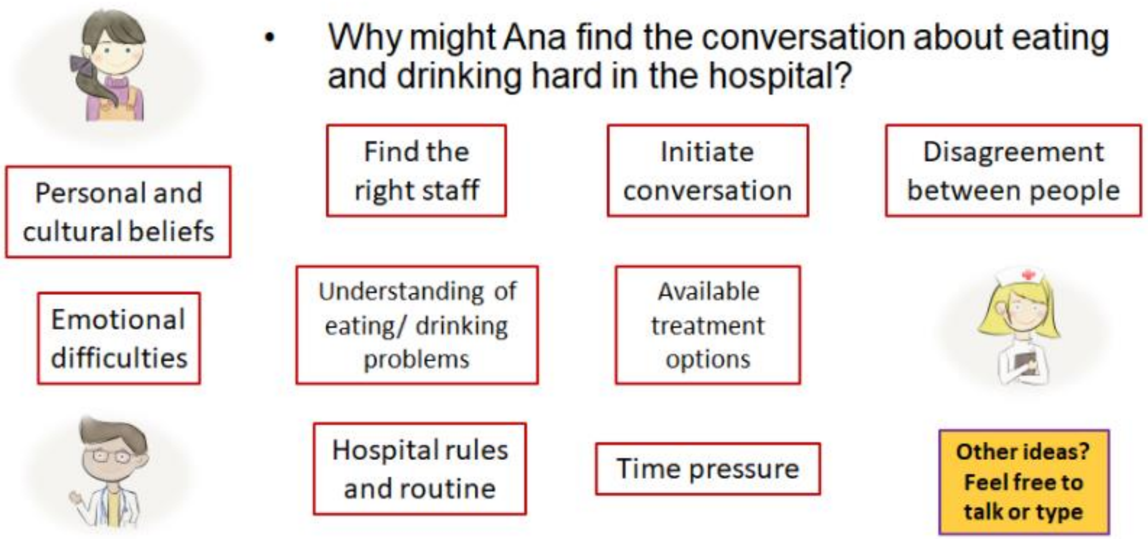

Voting!

Activity 2: How to '**initiate**' the conversations in hospitals

- What would help Ana when she starts a discussion with hospital staff about John's eating and drinking problems?
- How can the hospital staff know what to ask or explain to Ana?

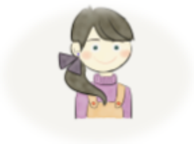

Conversation starter

Who and When

Level of understanding & information needed

Somebody to speak to

Badges for the staff -so we know

Availibility +/- all the time

Attitudes of the staff

One, consistent person across the admission

Understand about the role of each professional

Signs on the board/bed - aware of dementia and eating and drinking

Checklist

Clear pathway - know the route

What the options are

Use it in your time, pacing

More interactive - booking, online

Safe space - not in front of the person

Activity 3: How to '**engage**' in the conversations in hospitals

Listen to carers input

- How can we make sure Ana is involved in the discussion about John's care?

Set of information & questions

Two-way communication

Emotions

Trust

Disagreements

Personal beliefs

Time & space

Not systemic

Leave some contact details

Clear, one point of contact

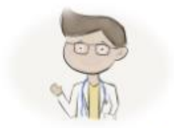

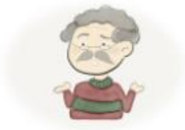

- How can we know if Ana is ready to finish the conversation?

Feedback - continue with questions&feedback

Have some time to digest things and come back with Q

Empathise what we have been through

Avoid repeating the same things (problems)

Body language and friendly interactive

Food allergy, somethings overlooked

social assumption

Not patronising

language - communication

Examples from the second workshop with hospital professionals

Activity 1: Decide on the aims of our guide

Being clear who would need some options (e.g. tube feeding) , but the others don't or not appropriate - what conditions and stages of [illegible]

- What are the specific aims that our guide can address?
- What are the outcomes that family carers and hospital staff may expect when using our guide?

Explain gentley about dementia being a terminal condition

documentation about palliative approach

Try to bring about the personalised plan - working with them

Background information

Help walking through the conversation

presentation of eating and drinking problems

clear, clarity

Simple language

Focus on the issue, may not too much on option

Staff worried saying things

Shortness vs too many things

Initiate the conversation

Get people to talk more

Some behavioural challlenges and also address certain types of dementia e.g FTD

Avoid general things - maybe not everyone needs it

Different options - keep just important points

Also about treating acute illness

Short term use

Medical team decisions - may not really family decisions

Patient care centred

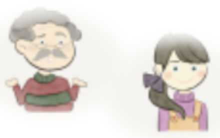

Conversation focused

Individual level

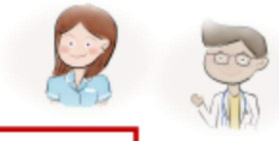

Staffing resources

Government & hospital policy

some terms can cause conflicts - like best interest, medical decision-making

Activity 2: What do you think about the initial draft of our guide?

Present the tube feeding in more abstract definition, may not how to discuss with carers

Prompts to improve a decision discussin to decision-making

Conversation starter

Open up all the options - could lead them to think they can choose all

VS think of booklet - space people can jot down

- What are the **pros** and **cons** of the draft?

(Suggestions to improve)

Emphasize that it's everyone's business

Personal autonomy vs best interest according to MDT

Think about the average reading age - use plain English

folding leaflet - easier to follow

Maybe

About the repeating things

try to use simple language, now there are technical terms

Having two versions - framework about decision-making + next more details

Like the quesions to ask

Use of picture more

Stressed situation - people don't want more reading materials

Check how to describe risk feeding

Check if repeating things

Shared information, building trust - people having read the same things

Try to build trust

The recognition of the importance of eating and drinking

Emphasize why the decision/ conversation is an issue

The current version is too busy

Possible tactics

Q&A format is good now

Why this is giving to you - not everyone wants this guide - explaind why this is for you

Fears of legal process - some would do what family wants

Problems related to dementia - introduce that not only memory

Mindful about the wordings - tweak to not stress one way or another

Older carers may have vision problems - size and colours

One page for steps for conversation then booklet to support Q&A

Balance between giving all information or some important information and help more on decision-making/conversation

Have a think about the term 'risk feeding' and 'comfort feeding'

sips and nibbles

Hints: You may think of our guide to be used in hospital settings.

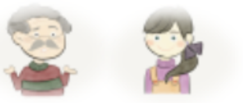

Indicate that the tactics not precriptive, more things can be done

Eating and drinking with accepted risks
