# Supplementary material for: Co‐design development of a decision guide on eating and drinking for people with severe dementia during acute hospital admissions
Source: Health Expect. 2023 Jan 17;26(2):613–29. doi: 10.1111/hex.13672 (PMC10010093; doi:10.1111/hex.13672)
Supplement: Supplementary file 4 — Supporting information. [file HEX-26--s006.pdf]

File S4: Completed data in WS4 Matrix table

Matrix for synthesising data and developing the decision guide

Key components	WS1 Systematic review Decision-making process	WS2 PLWD (mild) interviews	WS3 Carer interviews	W3 Professional interviews	WS4 Workshop and meeting discussion
Common admitting conditions	NA	NA	Falls, fractures, UTI, stroke (TIA), severe dehydration, heart disease; recurrent constipation	Infection, falls, delirium, renal failure, stroke, behavioural problems	Falls, stroke, infection, fracture, dehydration
Common eating/ drinking problems at home or in care home	Inability to recognise food, food refusal behaviours, chewing and swallowing difficulties, recurrent chocking and aspirations, change in alertness, significant weight loss	Most PLWD are not really aware of eating and drinking problems resulting from dementia; appetite and preference changes, social eating; their choice to change the habits or preferences to maintain their health and hope to prevent the progression	Unable to recognise food or utensil, forgetfulness, behavioural problems, just stop eating and drinking, very hot weather then dehydrated; poor eating and drinking care in some care home; constipation and incontinence issues; may not be aware that pre-existing problems are related to dementia	Baseline eating and drinking problems; changing preferences of taste or texture; history of recurrent chest infection suggesting existing swallowing problems; some carers might do very well and intuitively adapt the eating routines – may encourage them about this	Poor intake at home or care home; ask about eating and drinking patterns at home
Common eating/ drinking problems in hospitals	Acute illness vs the progression of dementia	NA (community settings)	Eating and drinking less; unattractive hospital food; cultural food; nobody stays with PLWD; unable to reach food (broken arm); positions in bed; unfamiliar environment and staff; distraction; swallowing (chocking); constipation, incontinence	Eating and drinking problems resulting from acute illness e.g. delirium; decreased awareness; waiting for operation; not their routine; unattractive hospital food; eating and drinking at EoL; positioning; hospital turnover	For (family) carers, not having someone to encourage eating and drinking; unattractive hospital food; food menu; not knowing information about nutrients of hospital food and what PLWD should receive; not only eating less but also other eating problems like eating weird food or eat too much; Meeting – may eat strange food -variety of sugar
Identify the problems and decisions	Whether and how to start or forgo ANH, to continue or stop risk feeding, to consider risk feeding; checking mental capacity, other causes e.g. depression	If any, focus on appetite and preference changes, social eating; resulting from their choices or circumstances; not knowing physiological changes, e.g., swallowing in moderate and severe stages; avoid social eating (other	To offer favourite food and drink; some already had eating and drinking problems at home and carers want to pass the information; some don't have a chance to stay and monitor; limited time as PLWD were rushed into acute care; think staff can't see PLWD individually; staff	To nil-by-mouth - sometimes to wait for assessment by SLTs; to use ANH (NG tube or IVF) to bridge the gap of acute deterioration; nurses and doctors activate further assessment and MDT meeting; if the family find food left uneaten in hospital, it's rare	For carers, want to know how their relative is doing in the hospital, but staff are very busy; check if eating and drinking is the problem and how; the staff may not have time to look after PLWD as an individual; sometimes they don't recognise that the individual has dementia; *'not only in but also out'*; good to have someone sit and look if eating problems; wonder if the admitting reasons cause the

Key components	WS1 Systematic review Decision-making process	WS2 PLWD (mild) interviews	WS3 Carer interviews	W3 Professional interviews	WS4 Workshop and meeting discussion
		might notice changes, gently excluded)	may assume PLWD can eat normally and don't need support; family may feel shocked or worried if they discover that hospital does not discuss with them before changing eating and drinking support to their relatives; COVID further minimise the chance to discuss	that they don't raise this to the team, review notes and check capacity of PLWD first if not then seek for family; confusing hospital environment and rotating hospital staff may cause eating and drinking problems; multidisciplinary help identify problems and share information	eating problems; eating and drinking might be new things for some carers; For staff - some staff might not regularly see the food, fluid and bowel chart – clear roles, but not for food things – don't get the whole picture of food and drink problems; constipation cause discomfort; asking family carers about eating and drinking at home could be difficult as they might not recognise if some changes are the problems
Initiate discussion or conversation	Day-to-day decisions (with lacked support) vs shared decision-making approach; acute condition may think of ANH; VSED requested by PLWD is challenging if he/she still accepting food/drink; nurses (+/- family) identified and informed problems but later excluded from decision-making	Prefer to leave discussion until problems occur; some acknowledged their future deteriorating ability to discuss;	Most carers confident to talk as they have been through many difficult discussions; but thought others might not feel assertive; wait for staff to approach; some not having a chance to discuss (partly due to COVID); some staff did not ask family about advance directives and provided care against the directives; most want to discuss in private and safe space, not in front of PLWD; each professional would have different role, so want to know why they are involved and who carers can talk to; process that staff take history before going to the family may be seen as a delay and cause confusion to the family	Try to communicate and simplify things to PLWD; check if there is ACP or stated wishes and preferences; some staff would ask permission from PLWD before going to the family (maybe informally); then write recommendation or bring to MDT or best interest meeting; specialities try to get to a physician in charge to keep on the same page; most want to involve the carers; some specialities may have been under-recognised and difficult when approaching to carers e.g., SLTs and palliative care team; may need to address acute illness first and sometime it's not eating and drinking	For carers – who speak out could be seen as 'problematic'; staff's verbal and body language; some young carers reported age-biased attitudes of staff; very difficult for carers to find someone to talk to; staff look very busy all the time; consistent point of contact; book time to discuss; a badge on staff who they can talk to about food and drink; want to know why the staff involved e.g., SLTs, palliative care team; For staff – go to PLWD first, even unable to decide but if any signs of accepting or refusal will then be respected; really depend on who family goes to speak to first; HCAs might not be the individuals who can directly talk about this; the conversation can happen anytime 24 hours; need to check if the individual is the main carer or LPA; some families might not have thought of having this conversation – levels of preparedness;
Exchange information: *understanding disease and interventions*	Family and HCPs can have poor understanding and unrealistic expectations of tube feeding; if PLWD not immediately dying may consider ANH; most important factors – poor outcomes and	Most participants were uncertain about their health and what eating and drinking they would have at later stages; also, see the row below	Most not really understand eating and drinking; want to know risks and benefits of each option; some think of past experiences of people who needed help with feeding and ANH; few worried about maximising nutritional completeness	Assess baseline – try to check overall progression of dementia and eating/drinking problems at home; see where PLWD is in their journey; also, seriousness of acute illness; then they can give recommendations properly; some other staff might focus	For carers – some found information around eating and drinking at the later stages and EoL very confusing; said often overlook food allergy, constipation; staff may ask about the previous admission – what improved or worsen; Staff want to get collateral history as much as possible; understand what would need for nutrition or not harmful; not every staff members really understand the options;

Key components	WS1 Systematic review Decision-making process	WS2 PLWD (mild) interviews	WS3 Carer interviews	W3 Professional interviews	WS4 Workshop and meeting discussion
	irreversibility in advanced dementia			only on treating acute illness; need to find the right family member to speak to (who knows the individual best); some mentioned difficulty with managing family dynamics as part of decision-making process; limited time in hospitals, so MDT would help assess the individual's history	difficult to get a whole picture of eating – different roles; knowing levels of information needed by really listening to the carers, unpack what they understand; communication skills; things might change – build upon the previous conversation or understanding; good for the carers to understand dementia being a terminal disease and involve other aspects, also not only one eating problem
Exchange information: *explaining disease and interventions*	Insufficient and biased information if HCPs think any feeding could not be forgone; fear of aspiration and legal litigation; think about individual's life history, age, past experiences, wishes and current wellbeing of PLWD; timing to explain things to family	Want HCPs explained risk and benefits and helped with decisions esp. if being treated with acute transient illness or approaching EoL; think of past experiences of relatives needing the interventions; if so, be familiar or accepted them including risk feeding, ANH	Want to inform hospital staff some basic things but can be overlooked – glasses, hearing aids, eye drops, dentures, medication regimen; explain to staff what would help for their loved one (what they have tried); want to help communicate for PLWD; but still want to listen to staff's recommendation; want to know if ANH is just for short- or long-term; want staff to unwrap different scenarios; honest and clear information; there is fragmented and poor information sharing in hospitals, so they have to talk to many unfamiliar staff (responsibility place on family to share the information); most family feel they don't get sufficient and timely updates about their relatives during the hospital admissions, including about eating and drinking; many may want HCPs to help make the decisions as they would know best, but the family still wants to know complete information	Some carers would not be ready to discuss – need to check their readiness and how much they want to discuss; need to explain all the consequences if carers inclined to tube feeding; explain mechanism of eating and swallowing; normalise that when people get very sick (like flu) they don't want much food or energy; also EoL; eating and drinking is natural process and people may not aware of it until the problems occur – so it's difficult to explain – but link to the natural mechanisms of eating and how dementia could affect the mechanisms would be helpful; try to simplify things and build on what the family says; for palliative care team, they can't just jump in but need to sensitively explain why they are here – which may require long discussion; it's difficult as they also need to be sure to some extent about the end-of-life of the individual	Carers need time to digest information and come back with feedback and questions; want to know all the options first then the pathway of each option – pros and cons; don't want to repeat saying things to different staff (not get into solutions, but the problems); want to know EoL signs; checklist where are we with the patient; carers' insight into the behavioural aspect vs staff knowing the clinical aspects; static information; For staff – find it difficult to explain or discuss death and dying process and often left to clinical team; food chart can help explain patterns; getting wrong information in the first place, esp. ANH, can make things worse – set the tone of the next conversation; family can only hear and remember the negative information; might not be done in one session – family can go home and chat with others; try to engage and educate family at the same time; may not be resolved at hospital level – then phoned up the family everyday; easier to talk about tube feeding and risk feeding in a more abstract way, rather than technicalities – will raise unrealistic hope; some specialists may not necessarily be involved – if putting in the guide the carers may question about the care; Meeting – inconsistency in explaining 'risk'

Key components	WS1 Systematic review Decision-making process	WS2 PLWD (mild) interviews	WS3 Carer interviews	W3 Professional interviews	WS4 Workshop and meeting discussion
					feeding – sometimes eating for pleasure, comfort feeding
Acknowledge emotions of all involved	Family needs emotionally unable to assess information and need more time; some requested PEG even were suggested otherwise; Uncertainty around disease progression, lack of knowledge, confusing roles lead to feelings of guilt, exclusion, frustration and conflicts; regular, open discussion, team approach, build trust; may call for ethics committee	Unsure about hunger and thirst at the EoL; don't fear of 'naturally' dying but of living with poor conditions, being no longer themselves and burden to family; want their family carers think about their own feelings and wellbeing when making decisions	Difficult but they have been through more difficult situation and discussion; quite ready if staff want to explain things and discuss with them; food and drink has a unique meaning to the individual and their family; in acute hospital, family carers often feel frustrated because poor communication with clinical team	Most recognised this is emotive discussion for carers; younger staff may find it more challenging – more experiences will get better; but some hospital staff still feel (subtle) unprepared every discussion; many staff feel reluctant to involve palliative care team; staff perceive many family might not be ready to discuss about palliative and end-of-life care; they need to reassure the roles of palliative and there would still be care plan in place for the individual	For carers – confusing information so feel unprepared for eating and drinking problems, especially at the later stages; feel frustrated as sometimes staff not recognising their carer roles and experiences; want staff to know emotional and spiritual experiences; well-being of carers often overlooked – the patient is the focus; For staff – think it depends on how the family has presented – agitated, distressed; some (inexperienced) staff who don't know about the options might not be confident in presenting the options – hesitant; feel 'getting things wrong'; difficult to have conversation around death and dying; fear about how the conversation might end; fear of saying things; some family might not have thought about this before; monitor family's emotion and stop when it becomes too much; family may fear of not knowing the whole things (trust, something kept secret)
Clarify values of eating/ drinking *problems and interventions*	ANH as basic human care rather than medical intervention in some countries; withdrawal more difficult than withhold ANH (more concrete to death); AH more acceptable than AN esp. in acute illness; social, religious, racial, professional values	Eating/ drinking as means of staying healthy; want to maintain autonomy – choose or cook food; fine with losing weight (always on diet); techniques using with children (coaxing, aeroplane) represented role reversal and being treated like a child; ANH as unnatural, not bring enjoyment and QoL; ANH would be useful for others with dementia but not for themselves; AH more acceptable esp. treating acute condition (don't want to die prematurely);	In some culture eating and drinking is very important as the way to provide care and show love; cultural food and drinking (sometime very specific type of food) – some PLWD may find traditional hospital food not attractive to their relatives; less flexibility of hospital food menus, mealtimes; want to bring food and drink from home, which sometimes are their cultural food; try to keep PLWD comfortable and good QoL, and not doing anything invasive; most family want to help the individual continue enjoy eating and	Acknowledge that food has meanings in it; some family would want to keep offering food; some provide cultural food on menu but may not have variety as limited by the hospital; it can be difficult to be culturally sensitive; meals from family can keep up to 24 hours and regulations around safety + microwave use; try to explain eating patterns and energy needs at EoL; generally oppose to ANH in advanced dementia; think about QoL; PEG is not indicated for severe PLWD; different wards and	For carer, food as fundamental issues of life; they know the individual best, including for eating and drinking; For staff food has meaning, symbolic more just than to provide nutrition – love – ask what they like to eat and how; first learnt as a child; more difficult to withdraw compared to medications; need to understand cultural difference but also be careful with the wording in order to not alienate anyone; language and terms like starving to death; good enough intake when PLWD are very ill – unrealistic expectation; not giving ANH, not starving to death; if anyone is pushed towards certain choice like ANH

Key components	WS1 Systematic review Decision-making process	WS2 PLWD (mild) interviews	WS3 Carer interviews	W3 Professional interviews	WS4 Workshop and meeting discussion
			drinking by mouth; ANH is take away part of someone's life; different wards would have different policy and approach to support eating and drinking problems; and some wards are not really keen on caring for the individual with dementia	expertise would have different approach to support eating and drinking problems; it's important that who is the first staff member that family would talk to as it will affect the following conversations	
Clarify values of approaching the *decisions*	Respect autonomy of PLWD; ACP about eating and drinking were rare, but honoured if available; ACP can be vague, outdated; if no previous wishes made, family and HCPs relied on presumed wishes, interpretation of current behaviours (appearing to decline) – challenging to understand; if all unclear then family's values and social norms would override the decisions	Anticipate gradually loss memory and decisional capacity; accept to gradually transfer responsibility to family but still wanted HCPs to give recommendations; still want to maintain agency as long as possible and be respected; few views in advanced dementia they would not feel distressed or mind what people offer (less awareness); home always best place to live/die; if PWLD refuse to eat, it could mean they don't want to stay alive	Help communicate what PLWD likes or wants; want staff to consult them as they know PLWD best; some would speak until they're heard, but others might find it difficult; involve other family members only if there is really involved in the care; if the death was not imminent, it is important to provide adequate food and fluids to get them well and improved from the acute illness; some would think if the individual stops eating, it's the sign of dying process	Some staff think ANH is medical decisions, they will only discuss and get inputs and advice from family; it's illegal, unethical and unfair to let family decide, except there is health LPA; 'how could they know'; think about overall progression vs acute illness; everything always needs to be the person-centred; may involve palliative care team; if many family members, just talk to someone who has been with PLWD; sometimes decisions can be delayed; sensitively explain things to their culture and language; it's very important to build up relationships as this will affect the decision-making process	Carers think it's the best to communicate on their level of knowledge instead of what you want to communicate; want to know sign of EoL for eating and drinking; carer know the behavioural aspect; don't talk down to people – they may know about these things; For staff – prefer to reach PLWD first, check if they have capacity or express what they like; some staff might need to pass the decision to more senior staff – then they don't gain their experiences; good to emphasise this may involve many different experts and many people may come to talk to you; some technical terms may cause conflicts like 'medical decisions' or 'best interest'; Meeting – need to explain terms like 'risk' sensitively and focus on quality of life
Consideration of feasibility	More severe problems may require more time and staff which should be equally distributed among patients in healthcare settings; in some settings use ANH to limit cost + prevent unnecessary hospital admission; some family perceive ANH is to make it	Wanted the family to think about their own wellbeing and ability to provide care; don't want to burden and restrict the carers' life; some consider moving to a care home to receive professional care and surrounded by people with the same condition; few views that	Hospital routine and meals; happy to be there and help eating and drinking; but for some PLWD might not have relatives come in; limited visiting time (especially during COVID); busy hospital staff to support each PLWD; unsure about immediacy to life and death; different wards have different skillset and rules; some	'Ideally' everyone come in the same room and discuss the decisions; difficult to be at the right time to speak to physician in charge; leave medical notes and recommendations; different professionals, different approaches; staff shortage; decisions over the weekend; need to see other	For family carers, different wards may have different practice around care for eating and drinking (preferred dementia ward); staff shortage; strict routine and hospital rules; For staff, there are quite a lot of rules of what you can have for example, alcohol – then hospital food not resemble what they normally have at home; might need to loosening the visiting time – what fits one person might not fit the hospital team; it's important to be clear that

Key components	WS1 Systematic review Decision-making process	WS2 PLWD (mild) interviews	WS3 Carer interviews	W3 Professional interviews	WS4 Workshop and meeting discussion
	easier for HCPs to provide nutrition/hydration; some countries offered incentive to use of ANH and some long-term care facilities require PEG before transfer from hospitals – pressure to HCPs; also fear of legal litigation	ANH as the only options to avoid death from dying which is distressing for family; but not the ideal for themselves; some PLWD mention about euthanasia, but they know this is not legal in the UK	think about the best place of EoL care like hospice; safe and private room, away from PLWD; some family carers mention about euthanasia but are also aware of the UK legal context	patients in the same ward/ bay; do med round; appreciate help of healthcare assistants and family in helping eating and drinking; provide protected meal time; think about discharge plans if there is community support; multidisciplinary can help assimilate the history and plan	this is medical decisions but the carers' inputs are important; the guide may explain that this is not prescriptive – so open about what can be done, depending on where you are; Meeting – not every family can have people coming into the hospital and some would need to use their own budget for food
Communicate preferred choices	Family and HCPs often feel uncertain about PLWD preferences; HCPs had better knowledge and less worried about litigations advised against PEG; some family avoided discussion and left decisions to HCPs; unnoticed decision-making (few cases) if clear what decisions should be made, very severe conditions, clear agreement in place	Accept encouragement, being around, texture modification; don't like techniques using with children; most view it could be limited ways; general opposition to ANH; those accepting ANH seem not fully understanding possible problems and intervention; Wish the EoL to be comfortable, free from pain and surrounded by loved ones, and die peacefully	Some can speak firmly of what they want for PLWD; sometimes they have some conflicts with staff but continue advocating for PLWD; preferences normally come from their care practice at home (what have been working); some prefer to discuss away from PLWD	Staff often go to PLWD first to check their preferences; allied team (SLTs, dietitian) would give recommendation (both professional and personal view) but then it depends on clinical team; some think of their own caring experiences of family; physician and nurse may display information to align to PLWD's best interest more than to family's preferences (person-centred approach)	Carers might not understand the situation/ options, so can't communicate their preferences; if carers are confident, they might be seen as 'problematic'; want consistency in communication and their message passing on; For staff, it needs to talk someone from many family members – the main carers avoid (re)start from scratch
Make a final decision and communicate the decision	Physicians usually are final decision-makers; but sometimes family; conflicting opinions of multiple family members resolved by law or closeness of relationships; non-physician HCPs and family are excluded esp. in paternalistic health system	Want family to make decision with support from HCPs; but still want carers to respect their wishes if they were requesting certain food or strongly refusing; prefer risk feeding at the EoL	Willingness to involve in the decision may vary; but most would rely on HCP's opinions as HCPs would know best about the condition and options; still want HCPs to listen or consult them; but always want full information; may discuss with other family members, but decisions would be from them who are the main carers	Some staff think is' unfair to push the decision to family – too stressful and unethical (and unlawful); some SLTs and dietitian think it's clinical team's decision (they just give recommendation); physician would be the decision-maker; some take charge as it's legally bound to medical decision; sometimes need MDT or best interest meeting which most find it helpful (but not always happens); most decisions can be waited – but avoid	Carers need time to make up the decision – can't do if it's too tense; want to be consulted; sometimes it's medical decision because it's too much for family but still need inputs from family; For staff fear about where the conversation might end; record the conversation and decision in a form and pass on; the eating and drinking decisions are medical decisions, but using inputs from family or carers; for some staff – tube feeding is not their choices and would not bring it to the discussion (mild-mod would be okay, but not for severe dementia); but some staff thought it would be good to have all the options presented to the carers and be clear

Key components	WS1 Systematic review Decision-making process	WS2 PLWD (mild) interviews	WS3 Carer interviews	W3 Professional interviews	WS4 Workshop and meeting discussion
				unnecessary nil-by-mouth; it's important to reassure the family what have been done and now what's no longer effective or benefits to their relatives	that why it is inappropriate (people would see it from other beds anyway and ask later)
Provide eating/drinking interventions	Day-to-day decisions need immediate actions; accepted refusal but not over long period; used tricks e.g., reminders, soft touch with spoon to postpone ANH decisions; Shared decisions – HCPs agreed to risk feeding if family were well-informed about risk of aspiration and weight loss; ANH viewed inappropriate in long term (except in some countries); ANH accepted for temporary – part of treatment of acute conditions	Not exactly	If the hospital allows, they want to come in and help with eating and drinking; would bring their favourite, cultural food and encourage PLWD to eat and drink; sometimes need to remind staff to give food and water, especially if the PLWD was nil-by-mouth; most family carers don't want to force feed the individual (neither orally nor via ANH)	Try many possible ways; sometimes need trials and errors– temporary ANH (NG tube and IV fluids) then check if the individual has been doing well with eating and just recently changed because of reversible causes; healthcare assistants (HCAs) are valuable members who help a lot with eating and drinking and might know best how PLWD is doing with the intervention; some want to show the carers how they support - then they can do this at home; can help understand about eating and drinking process; often nil-by-mouth unnecessary as SLTs are not in over weekends;	Staff support the compassionate visiting – happy for the family to bring food in (difficult during COVID); want to fill in a food chart and have a look for the trends or patterns – think of what else should be offered – variety; PLWD may not eat with staff at all; family can teach the staff how to help eating and drinking at bedside – looking at their positions, speed and then replicate; food chart can help thinking of alternatives and variety of food and drink; Meeting – not every family can have people coming into the hospital and some would need to use their own budget for food
Monitor and evaluate the support	Ongoing evaluation until stabilised to resume oral feeding or wait for decision of long-term ANH; AN (few weeks) expected to be used longer than AH (days); ANH might require restrains leading to complications and distress; post-decisions – nurses usually provided further medical explanation, psychological support,	Fear of having battles at mealtimes from being coaxing; if they strongly refuse, they carers should stop	Want HCPs to closely observe what PLWD is doing and adapt the support to their needs; would stop or challenge HCPs if they find the feeding is not right for PLWD; sometimes difficult to know what's going on in the hospital – want more communication and regular updates	Allied team would not often follow up the decisions, but if things go wrong or not improved – they will be re-consulted and reviewed; if PLWD look unhappy and pull NG out then not want to push it back; decisions (and recommendation) should be documented and shared (e.g., safe swallow, accepted risk feeding to avoid nil-by-mouth)	Carers need contact details of the individual who take care of eating and drinking; may use some online technologies that can automatically send daily updates to family carers; For the staff, need to monitor both 'in' and 'out' (constipation can be very discomfort and affect eating) – try to reduce medications like laxatives; consistently record as soon as possible – forgetting if they ate or went to toilet; also check environment – too noisy; call families to inform would help them not to chasing for the staff; Meeting – patients may hide or forget food so hinder the food chart –

Key components	WS1 Systematic review Decision-making process	WS2 PLWD (mild) interviews	WS3 Carer interviews	W3 Professional interviews	WS4 Workshop and meeting discussion
	and/or preparation for death				but very useful tool; but towards the EoL – food chart may not be relevant – to keep QoL, not counting the food and drink
Renegotiate the decision	If not mutually agree – challenging opinions, questioning or refusal to enact; non-physician HCPs may personally influence family; sometimes HCPs had to act against their beliefs to provide ANH to follow family's request; some HCPs deliver ANH in more tender and respectful to minimise discomfort (mouth care, pain, position); if ANH's burden outweighed benefits then call for renegotiation – often stop ANH and start risk feeding	Our findings suggested that what PLWD says or does in the moment may not last and offers of food can acceptably be made later	Continue to ask or request until they get the right things for PLWD; if the PLWD entered the EoL, just wanted to keep them comfort and did not want to admit to the hospital again; some brought PLWD to hospice and let them die peacefully; most can let the eating and drinking go but mentioned about other people might not be okay due to cultures	May try to follow family if they resist but still focus the best interest of PLWD, not the family's wishes; showing how to provide eating and drinking support can help family understand more by demonstration; try to explain; if the tube comes out, blocked, unusable – it's the point of decision; need to (re)explain and think about bereavement – let family look back and feel they've tried everything possible	Carers want staff to come back and follow up; messages are not being passed on; remote conversation is good; For staff, family can only hear and remember the negative information like the individual is dying, not treatments are still going, so it needs to recheck their understanding and restate the important point from the last conversation; food chart can help visualise and discuss the prognosis and future plan with family, i.e., seeing the dying process; fear of how the conversation might end; but things can change, so important to have follow up conversation; can phone call afterwards
Post-discharge education and support	NA – but day-to-day decisions by family carers would still require post discharge support	NA	Most could not recall specific support from the staff; most thought it could be because of hospital environment then PLWD could eat and drink normal when getting back home; some hospital admission is short so this is the issue staff would focus; some wanted to know more about the progression of eating and drinking problems after this; some had SLTs coming to assess and professional carers to help with meals which were helpful; some were discharged to palliative care team in the community but later the PLWD lived for another 2 years (could	Do not provide the support to everyone; it is case by case basis; if it's required, then provided written information (letters, medical notes, leaflet) and phoned the community services to link up; also, to GPs; prevent readmission, e.g., to let the family or community know about risk feeding; It is important to get ACP done here (also to prevent readmission) but it is often that this is done quickly enough and people coming back; fragmented information sharing systems, especially over the	Carers think some service might be involved – need to know available resources in the community For the staff, try to put in notes or online system to pass on information which prevent hospital readmission; let the GP and district nurse know about the conversation help in hospital – like ACP; help the family practicing how to have the conversation – how to tell everyone else what has happened – also a tool for monitoring the understanding; provide written information -avoid going back and ask the same things

Key components	WS1 Systematic review Decision-making process	WS2 PLWD (mild) interviews	WS3 Carer interviews	W3 Professional interviews	WS4 Workshop and meeting discussion
			eat better and recover at home); it is also important to address other symptoms like behavioural symptoms as this will also impact the care and eating	weekend that some notes may not be handed; some specialists' recommendations are not included in the discharge summary; community services might not always be available	
Facilitators	Mutual support for family and HCPs; understand and respect in their roles, establish trust from open communication/ discussion; have a chance to renegotiation (time delay, trials and errors?)	NA	Carers generally used to have some more difficult conversation, so they were normally ready and prepared for the conversations; need the staff to be honest and give clear information; safe space and enough time; have contact details	Having more skills and experiences, becoming more confident to discuss; inputs from MDT could help difficult conversation like EoL; clear pictures of overall prognosis and baseline eating and drinking	Empathy, not patronising, value carers inputs; mutual respect; safe space and enough time to understand and think about the situation; information – feedback – back with questions then feedback (loop); booking appointment to discuss more details; regular updates and contact; understanding of nutrition for people with dementia; MDT helps the conversation and decision-making; staff engage with conversation and listen to carers rather than going through all the options; having something to get the conversation is very helpful (conversation starter)
Barriers	Unpredictable prognosis of dementia; unclear role and responsibility; limited time to make decisions; conflicting opinions; unreliable ACP; difficult to interpret behaviours of PLWD; social and organisational expectations or rules	NA	Lack of understanding about eating and drinking problems; hospital meals, mealtimes, and routine; environment; language and communication	Language, communication; multiple family members with different opinions; some staff were not open to the recommendations	Attitudes of staff towards carers if they are speaking for PLWD – problematic, younger (inexperienced); tense emotions can't think of decisions; no system to pass on information; pandemic (COVID); being admitted to mixed ward; involve GPs early on; lacked understanding of roles of the staff; Staff think some carers just like 'I know better/ I know all'; hospital routines; busy ward – any records might not get updated; Meeting – main problems are to have continued conversation, record and pass on the discussion; signs over the bed can be ignored quickly, not updated and people move around the bay/ward and the sign left there; information can be kept with family and used when talking to staff;
Decision guide: format	NA	Having some difficulties in think of others' situation	Laminated; it can be online; a short document	Should be short (no more than 2 pages of A4); transparent –	See the last three rows below

Key components	WS1 Systematic review Decision-making process	WS2 PLWD (mild) interviews	WS3 Carer interviews	W3 Professional interviews	WS4 Workshop and meeting discussion
		(vignettes) but some can engage and discuss further		people can see both carers and HCPs version;	
Decision guide: techniques	NA	NA	NA	NA	See the last three rows below
Signpost additional information	NA	NA	NA	NA	Alzheimer's society website, UCL Nutri-Dem resources (see Section 3.9); signpost to further information - reference to it – if you have an issue with X talk to Y; available resources in the community
Other components	**Determinants**: decision-makers tend to forgo ANH if well-informed of risk/ benefits, recognise poor prognosis of advanced dementia, know the patients' wishes to stop feeding, have negative experiences of relatives, viewed ANH prolongs life unnecessary; ANH indispensable if unsettling emotions of family /HCP, focus psychosocial benefits of ANH, concern litigation, pressure from others to start ANH	NA			Carers want to involve GP earlier – need to flag this individual has dementia and needs special care; check grammar and use of abbreviations; For staff, national level of any guides, not only the trust level, would make the staff and family confident to use it; electronic system that link up all services across region or country like 'coordinate my care' in London; Meeting – this can be helpful for senior staff to use for teaching and training younger, less experienced staff about the conversation;
**Decision guide: WS3 workshops and research advisory team meeting**					
Decision guide: aims & expected outcomes	Carers – help facilitate the conversation though the difficult situations, encourage the conversation to get started, acknowledge the live situation, sections to put new information and personalised input, help the input from the carers to be heard; inclusive language – right to the level of user; give basic information about eating and drinking and options; roles and responsibility pf who can help; signpost to more information and community services<br>Staff – improving communication and get the conversations started, get to talk				
Decision guide: format	Carers - good to have online format with regular updates of PLWD; can be put over the bed to pass over information; 'baby on board' badge to identify staff; sign over the bed to identify PLWD; group discussion (education) can be good for logistic reasons if hospital staff are so busy; live documents – unable to have all scenario but can be updated to address new scenario; space to put the individual's needs; clear language and not talking down to people – they may know about these things; emphasise about empathy; stepwise - great to see the next steps – now you've done x, y, z then what; cultural sensitive but careful with words that could alienate people; For the staff, shorter version for laminated sheet over the bed and longer for electronic version; Meeting – should be clear if we're talking about relatives, formal carers, distant carers – may target those who know best; having clear aim to support conversation but may be not to change				

Key components	WS1 Systematic review Decision-making process	WS2 PLWD (mild) interviews	WS3 Carer interviews	W3 Professional interviews	WS4 Workshop and meeting discussion
	issues related to higher level policy; to help engage the staff with conversation rather than going through all the options; may not giving all information but signpost; having the same version for carers and staff would encourage culture of trust – reading and starting the conversations with the same thing (the whole things not be held behind); may start the first section with decision-making framework then go with more information in the next page;				
Decision guide: techniques	Carers – may need something attractive and inviting 'This is for you'; be concrete and short – to the point; even have the paper but still need to talk to the right staff; use of colour can help separate sections and highlight important things; visual appealing – reduced text – but aware of using too much; nice to have things in blocks; not too pointy or checklist – can be free form; interactive part is good – not only reading the guide; being comprehensive but very simple; avoid overlapping; think of more sequential style – like board game but not straight line – different steps people can revisit; think about the orders – people should know all the options first then ask their consequences; not only how but also when; For the staff – start with why this is given to you; they liked the use of questions you may wish to ask, who can be involved, inform the common eating and drinking problem, the recognition of importance of food and drink, the support specific to a hospital – some would suggest to make some parts more specific to people with severe dementia; check and use the sensitive language and if easy to understand for everyone; the carers can be older people too – think of font size and colours; if want to include more information then it can be a leaflet but if it's just for conversation guide then it can be 2 pages of A4 but need to be more concise (to much information in the draft 2 – 05.08.21); think about reading average 9 years old;				

Abbreviations

ACP = advance care plan; ANH = artificial nutrition and hydration; AN = artificial nutrition; AH = artificial hydration; EoL = end of life; HCA = healthcare assistant; HCP = healthcare professional/practitioner; IVF = intravenous fluids; MDT = multidisciplinary team; NG = nasogastric; PEG = percutaneous endoscopic gastrostomy; PLWD = people living with dementia; SLT = speech and language therapist; TIA = transient ischaemic attack; UTI = urinary tract infection; VSED = voluntarily stopping eating and drinking; WS = workstream; NA = not applicable
