# Supplementary material for: Co‐design development of a decision guide on eating and drinking for people with severe dementia during acute hospital admissions
Source: Health Expect. 2023 Jan 17;26(2):613–29. doi: 10.1111/hex.13672 (PMC10010093; doi:10.1111/hex.13672)
Supplement: Supplementary file 5 — Supporting information. [file HEX-26--s002.pdf]

File S5: Feedback form for user testing

Feedback form: Guidance to conversation and decision-making about eating and drinking for people with severe dementia during hospital admission

Are you a former/current family carer or hospital staff? *(tick one)* ☐ Family carer ☐ Hospital staff

We would like to know what you think about the guidance you have just reviewed.

1. Please rate each section, by marking 'poor', 'fair', 'good', or 'excellent' to show what you think about the way the information was presented on. *(Free space for further comments below)*

Section	Poor	Fair	Good	Excellent
**Page 1 – conversations and decision-making**				
Reasons why the conversation can be difficult	☐	☐	☐	☐
Steps of the conversation and decision-making	☐	☐	☐	☐
Questions you can think of and may wish to ask (or explain)	☐	☐	☐	☐
**Page 2 – additional information**				
Eating and drinking problems resulting from dementia	☐	☐	☐	☐
Eating and drinking treatments that you may have heard of	☐	☐	☐	☐
Who can be involved in the care for eating and drinking	☐	☐	☐	☐
People with mild dementia's perspectives and needs	☐	☐	☐	☐
Some help that could be available in a hospital	☐	☐	☐	☐

2. Overall, the length of the presentation was *(tick one)*

☐ too long

☐ too short

☐ just right

3. The amount of information was *(tick one)*

☐ too much information

☐ too little information

☐ just right

4. I found the presentation *(tick one)*

☐ slanted towards using tube feeding for the person with dementia

☐ slanted towards eating and drinking with accepted risks

☐ balanced

5. Do you think we included enough information to encourage a family member and hospital team get the conversation started?

☐ Yes

☐ No

Comment: Click or tap here to enter text.

6. Would you have found this guide useful when making your decision about eating and drinking for people with severe dementia in a hospital?

☐ Yes

☐ No

Comment: Click or tap here to enter text.

7. What did you like about the guide?

Click or tap here to enter text.

8. What suggestions do you think we can improve the guide?

Click or tap here to enter text.

Adapted from:

O'CONNOR, A. & CRANNEY, A. 2002. User Manual - Acceptability Ottawa: Ottawa Hospital Research Institute.

Retrieved from: https://decisionaid.ohri.ca/docs/develop/User_Manuals/UM_Acceptability.pdf
