# Supplementary material for: Co‐design development of a decision guide on eating and drinking for people with severe dementia during acute hospital admissions
Source: Health Expect. 2023 Jan 17;26(2):613–29. doi: 10.1111/hex.13672 (PMC10010093; doi:10.1111/hex.13672)
Supplement: Supplementary file 6 — Supporting information. [file HEX-26--s003.pdf]

File S6: IPDASi v4.0 checklists for the decision guide in this study

IPDASi V4.0 domains	1. Qualifying Criteria (6 items)		2. Certification Criteria (10 items)		3. Quality Criteria (28 items)	
Information	**(5)**	Met	**(1)**	Met	**(2)**	Met
	The patient decision aid describes the health condition or problem (treatment, procedure, or investigation) for which the index decision is required.	Y	The patient decision aid shows the negative and positive features of options with equal detail (e.g., using similar fonts, sequence, presentation of statistical information).	Y	The patient decision aid describes the natural course of the health condition or problem, if no action is taken (when appropriate).	Y
	The patient decision aid explicitly states the decision that needs to be considered (index decision).	Y			The patient decision aid makes it possible to compare the positive and negative features of the available options.	Y
	The patient decision aid describes the options available for the index decision.	Y				

	The patient decision aid describes the positive features (benefits or advantages) of each option.	Y				
	The patient decision aid describes the negative features (harms, side effects, or disadvantages) of each option.	Y				
Probabilities	**(0)**	Met	**(0)**	Met	**(6)**	Met
					The patient decision aid provides information about outcome probabilities associated with the options (i.e., the likely consequences of decisions).	N
					The patient decision aid specifies the defined group (reference class) of patients for whom the outcome probabilities apply.	N
					The patient decision aid specifies the event rates for the outcome probabilities.	N

					The patient decision aid allows the user to compare outcome probabilities across options using the same time period (when feasible).	NA
					The patient decision aid allows the user to compare outcome probabilities across options using the same denominator (when feasible).	NA
					The patient decision aid provides more than 1 way of viewing the probabilities (e.g., words, numbers, and diagrams).	N
Values	**(1)**	Met	**(0)**	Met	**(1)**	Met
	The patient decision aid describes what it is like to experience the consequences of the options (e.g., physical, psychological, social).	Y			The patient decision aid asks patients to think about which positive and negative features of the options matter most to them (implicitly or explicitly).	Y

Guidance	**(0)**	Met	**(0)**	Met	**(2)**	Met
					The patient decision aid provides a step-by-step way to make a decision.	Y
					The patient decision aid includes tools like worksheets or lists of questions to use when discussing options with a practitioner.	Y
Development	**(0)**	Met	**(0)**	Met	**(6)**	Met
					The development process included a needs assessment with clients or patients.	Y
					The development process included a needs assessment with health professionals.	Y
					The development process included review by clients/patients not involved in producing the decision support intervention.	Y

					The development process included review by professionals not involved in producing the decision support intervention.	Y
					The patient decision aid was field tested with patients who were facing the decision.	Y
					The patient decision aid was field tested with practitioners who counsel patients who face the decision.	Y

Reference

Joseph-Williams N, Newcombe R, Politi M, Durand MA, Sivell S, Stacey D, O'Connor A, Volk RJ, Edwards A, Bennett C, Pignone M, Thomson R, Elwyn G. Toward Minimum Standards for Certifying Patient Decision Aids: A Modified Delphi Consensus Process. Med Decis Making. 2014 Aug;34(6):699-710. doi: 10.1177/0272989X13501721. Epub 2013 Aug 20. PMID: 23963501.
